# Improvements in protective measures in factories with acetylene hydrochlorination and ethylene oxychlorination techniques declined risk assessment levels and affected liver health status

**DOI:** 10.3389/fpubh.2022.1053300

**Published:** 2022-11-22

**Authors:** Yiwen Dong, Xingang Wang, Weijiang Hu, Hongying Bian, Xin Wang, Ning Kang, Feng Han, Siyu Zhang, Meng Ye

**Affiliations:** ^1^Department of Occupational Epidemiology and Risk Assessment, National Institute for Occupational Health and Poison Control, Chinese Center for Disease Control and Prevention, Beijing, China; ^2^Department of Occupational Health and Radiological Health, Tianjin Binhai New Area Center for Disease Control and Prevention, Tianjin, China

**Keywords:** acetylene hydrochlorination, ethylene oxychlorination, VCM, improvement on protective measures, occupational health risk assessment, fatty liver, other hepatic symptoms, fresh air requirement

## Abstract

Acetylene hydrochlorination and ethylene oxychlorination are the two most common methods of producing vinyl chloride monomer (VCM), which has been linked to liver impairment, hepatocellular carcinoma (HCC), and angiosarcoma of the liver (ASL) in occupational settings. However, whether and how these impairments could be effectively improved from workplace root causes has yet to be discovered. This study aimed to evaluate whether improvements in protective measures in groups Y (408 subjects) and Z (349 subjects) could have an influential impact on the alleviation of liver impairment by comparing risk assessment levels under several semi-quantitative models and results from liver ultrasound detection and liver function tests before and after the improvement. Importantly, significant differences in constituent ratio involved in parameters among age, length of employment, weekly exposure time, smoking status, alcohol consumption, and sleeping quality were found between Y and Z before improvement took place in 2020 (*P* < 0.05 or *P* < 0.001), and population distribution by gender between Y and Z was in a large homogeneity with differences in age and length of employment. C_STE_ involves ore breaking, acetylene generation, steam stripping, outward processing, and welding maintenance, was disqualified in 2020 compared to OEL, and was said to have declined to meet OEL requirements by 2021. Further, a negative correction of fresh air requirement and ventilation air changing rate with ambient concentration toward hazards in Y was stronger in 2021 than in 2020. Significant differences in risk levels in Y between 2020 and 2021 were found as ore breaking, acetylene generation, steam stripping, outward processing, VCM polymerization, welding, and repairing, decreasing to relatively lower risk levels in 2021 from the original ones in 2020 only under the semi-quantitative comprehensive index model. Abnormal rates toward other hepatic symptoms decreased in the majority of positions after the improvement, as referred to by alterations such as ALT, AST, and GGT. Overall, the effect of improvements on protective measures effectively reduced positions' risk assessment levels through ventilation enhancement and airtight strengthening, which further affected abnormal rates toward other hepatic symptoms, and alterations such as ALT, AST, and GGT were much more significant in Y than effect in Z.

## Introduction

As an essential chemical material, vinyl chloride monomer (VCM) is used mostly in the aggregation of polyvinyl chloride (PVC), a product that is extensively used in anti-erosion pipes, construction materials, and automotive parts (1). Currently, the global market demand for PVC keeps rising, with an estimated annual production of over 30 million tons in 2021 (2). Globally, acetylene hydrochlorination and ethylene oxychlorination are presently the major procedures for synthesizing VCM and PVC. The former has taken up more than 80% of the production share and ~40% of the capacity annually throughout the central and western regions of China due to simple crafts, low investment, and an abundance of materials in coal and calcium carbide (3). By contrast, the latter one is usually distributed in southeast coastal regions, as it requires imported ethylene, high-tech reaction equipment, and matched purification measures. As the world's largest production base for VCM and PVC, China's capacity in 2018 reached 23.53 million tons, and it is expected to reach 25.93 million tons by 2023 (4).

Given that the large-scale health conditions of workers occupationally exposed to VCM might not be so optimistic, some protective measures under recent circumstances still have room for improvement. According to the previous investigation, VCM and other identified hazards threatened workers' health status at relatively high concentrations in ambient workplaces due to volatilization from leakage of unsealed valves, open sampling ports, or noneffective ventilation, which increased accidental risks for acute poisoning and adverse effects under chronic exposure (5). In 2012, the International Agency on Cancer Research (IARC) classified VCM as a group I carcinogen based on evidence from animal and occupational epidemiological studies (6). Subsequently, sufficient evidence in humans proved that VCM caused ASL and HCC, according to the findings from two large multi-center cohort studies at PVC production plants in the USA and Europe (6, 7). In a European study, the risk of lung cancer among the 12,700 PVS workers in 19 VCM/PVC plants significantly increased with cumulative concomitant exposure to VCM (8).

Furthermore, IARC also recognized that workers who were occupationally exposed to VCM were simultaneously exposed to other hazards, indicating that more severe adverse effects might be developed through joint actions among hazards that were homogeneous in target organs, such as the liver (9). Meshakova et al. estimated that employees from several large-scale PVC production plants experienced prolonged exposure to relatively low concentrations of VCM and 1, 2-dichloroethane (1,2-DCE). Both of them predominantly affected liver enzymes, forming 2-chloroethylene oxide, monochloroacetic acid, and the conjugated metabolic product of thiodiglycolic (thiodiacetic) acid (TDAA), which tend to be mutagenic and carcinogenic (10). Particularly, workers from the VCM division were subjected to simultaneous intensive exposure to concentrations of VCM ranging from 2.0 to 14.6 mg·m^−3^ and of 1,2-DCE from 15.0 to 87.2 mg·m^−3^, while those from the PVC division were only exposed to concentrations of VCM ranging from 1.1 to 10.7 mg·m^−3^ (11). In addition to VCM, PVC was classified as a possible carcinogen (class 3) by the IARC. The inhaled PVC dust (in particular, with an aerodynamic diameter of <5 mm) may remain in the pulmonary interstitium for years, gradually releasing residual VCM, which may account for the neoplastic transformation of an epithelial cell. Due to the residual presence of VCM and other additives, the European Union's Classification, Labeling and Packaging (CLP) Regulation reports that PVC is one of the plastic polymers with the highest health hazard (hazard score of 5) (12).

In this regard, workers exposed to VCM and PVC at workplaces are facing adverse health effects. Implementing occupational risk assessment in advance would be indispensable and urgent for identifying hazard factors and promoting liver function status. Recently, the methodology toward occupational health risk assessment (OHRA) has been well-rounded for risk assessment through several available quantitative or semi-quantitative models, including the Environmental Protection Agency's (EPA) quantitative model for carcinogens or non-carcinogens, the Singaporean semi-quantitative model, the United Kingdom's Control of Substances Hazardous to Health Essentials (COSHH Essentials), the Romania risk assessment model, and the International Council on Mining and Metal's (ICMM) quantitative model (13). Based on different models above, China formulated its own technical guideline for the occupational risk assessment model for chemicals in the workplace (GBZ/T 298-2017) from its predecessor, the Singaporean semi-quantitative model (14).

Thus, this study aimed to achieve several research purposes, including (1) systematically evaluating the effectiveness of improvements in protective measures in factories with different technological processes by comparing external concentrations among identified hazard factors before and after improvement and finding out engineering protection factors that might relate to the effectiveness if it works; (2) observing possible alterations toward risk assessment levels of VCM exposed positions affected by improvements on protective measures and comparing differences in methodology among three semi-quantitative risk assessment models; and (3) analyzing possible contributing factors that involve abnormal symptoms and morbidities on liver ultrasound detection and the liver function test. To the best of our knowledge, this could be the first study to emphasize occupational risk assessment of VCM-exposed positions in factories with techniques of acetylene hydrochlorination and ethylene oxychlorination before and after improvements on protective measures, which will pave the way for guidance implementation in occupational health surveillance and health management.

## Materials and methods

### Study design and subjects

A cross-sectional study of a PVC factory with the acetylene hydrochlorination technique in Tianjin City (Y) and another VCM synthesis factory with the ethylene oxychlorination technique in Guang Zhou City, Guang Dong Province (Z) was conducted in July 2020, right before their annual overhaul for repairs and maintenance (which usually takes place in November and would last for 1 or 2 months until early next year). Another retrospective investigation was carried out again in 2021 for alteration observation.

Concretely, 408 subjects from Y and 349 from Z who were occupationally exposed to VCM or others were recruited based on the following inclusion criteria: (1) employment duration longer than 1 year and longer than 3 months from current positions, (2) aged 20–55 years without gender difference, (3) work content involving operating or patrolling patterns for a certain period, (4) participants with complete questionnaire inquiries and physical examination data, and (5) workers with no medical history of allergy, asthma, allergic rhinitis, cardiovascular diseases, viral hepatitis in B and C, liver cirrhosis, or malignant liver cancer. The ethical approval of this study was approved by the Medical Ethics Committee of the National Institute of Occupational Health and Poison Control, Chinese Center for Disease Control and Prevention, Beijing, China (NIOHP202007).

### Questionnaires

All subjects who voluntarily joined this study were informed about its purposes and were requested to participate in a face-to-face questionnaire in July 2020. Precisely, parameters such as gender (men/women), age (~35, ~45, and ~55 y), length of employment (~5, ~20, and ~35 y), working shift system (8-h dayshift, 8-h shift, and 12-h night-shift), weekly working time (~40 and ~60 h), weekly exposure time (~20 and ~40 h), smoking status [(smokes at least one cigarette per day for 1 year or more, including those who have quit smoking in <1 year), (yes/no)], alcohol consumption [(consuming alcohol more than three times per week and more than 60 g/day each time), (yes/no)], sleeping duration (~4, ~6, and ~8 h), sleeping quality (good, general, and bad), conscious ventilation effect (significant, ordinary, and negligible), individual protective masks (always wearing, sometimes if necessary, and never), and status of ventilation installment (normal operation, temporary suspension, and fully broken) were collected. All interviewers were trained in advance for objective inquiry and content integrity.

### Physical examination data collection

The occupational physical examination was collected from the CDC of Binhai and Dagu New District of Tianjin City, in which the hepatic function index of alanine aminotransferase (ALT), aspartic transaminase (AST), glutamyl transpeptidase (GGT), alkaline phosphatase (ALP), and the serum lipid parameters of total cholesterol (TC) and triglyceride (TG) were included. The results from liver ultrasound included but were not limited to fatty liver in mild, moderate, and severe grades, multiple hepatic cysts, intrahepatic calcification, thickened echo, multiple gallbladder stones, cholecystic polyps, and chronic cholecystitis. They were roughly divided into categories of normal, fatty liver (mild, moderate, and severe), and other hepatic symptoms for analysis.

Specifically, a total of 384 out of 408 workers (94.1%) from Y participated in the annual physical examination in 2021, compared to 393 in 2020 (96.3%), with a slight decrease of 2.2% in attendance rate. On the contrary, 324 out of 349 workers (92.8%) from Z joined this activity in 2021, compared to 327 in 2020 (93.7%), with a decrease of 0.9% in attendance rate. The missing participants might be due to job transfers, retirement, or rehabilitation.

### Occupational on-site surveys

#### Identification of occupational hazards

According to an on-site survey, major occupational hazards in Y involved VCM, PVC dust, CaC_2_ dust, NH_3_, Cl_2_, HgCl_2_, HCl (36–38%, pH <2), NaOH (3.5%, pH = 13.9), welding fume, O_3_, manganese, and its inorganic compounds. Major hazards in Z were identified as VCM, 1, 2-DCE, Cl_2_, HCl (36–38%, pH <2), and NaOH (3.5%, pH = 13.9).

#### Status of protective measures before improvement

Protective measures in Y largely relied on the general ventilation effects and local dust removal, as many facilities were placed indoors, while measures in Z mainly depended on natural ventilation and airtight equipment, as most of its facilities lay outdoors. However, the effectiveness of protective measures for both of them still needed time to improve. In Y, problems such as insufficient emergency ventilation installation, malfunction on the part of ventilation equipment, irrational indoor air distribution flow, improper setting of exhaust hoods, absence of sprinkling and spraying devices, inefficient bag dust collectors, uncovered observation ports, unsealed valves or cover plates, fume cupboard malfunction, and so on were found to be potential risk factors for adverse effects. In Z, problems primarily concentrating on unsealed sampling devices, unsealed sampling ports, non-standardized settings on emergency rescue facilities, shortages of personal protective tools for certain positions, and a shortage of engineering protection measures during the loading and fueling process were potential risk factors for adverse effects.

#### Status of protective measures after improvement

After improvement, in Y, the raised dust of PVC and CaC_2_ was suppressed mainly by the installation of anti-dust fences, bag dust collectors, and sprinkler facilities; welding fume or other hazards stemming from maintenance and repair work were effectively expelled through local exhaust fans or draft fans; and facilities such as emergency ventilation, exhaust hoods, dust collectors, and axial defective flow fans were fixed up and put to use after tests and evaluation. In Z, most pipelines, valves, observation ports, and sampling devices that originally existed at the risk of leakage had been renovated by replacing old ones with highly efficient sealing and anti-corrosion materials.

### Sampling and detection

Occupational hazards were mainly categorized into two kinds: industrial dust and chemical hazards. According to Table 1, sampling and detection were conducted according to the standards of (15) GBZ 159–2004 *Sampling Practices for Monitoring Harmful Substances in Workplace Air* and (16) GBZ/T 300.1-2017 *Measurement Methods for Toxic Substances in Workplace Air, Part 1: General Principles*. In this regard, the sampling process was operated at representative sites at different time intervals and continuously sampling for three working days to ensure different individuals in identical positions were covered. Particularly, chemical hazards concentrations that related to maximum concentration (C_M_) were Cl_2_, O_3_, HCl, NaOH, and H_2_S, referred to as short-term exposure concentration (C_STE_) were VCM, PVC dust, CaC_2_ dust, NH_3_, welding fume, manganese, and inorganic compounds, and 1, 2-DCE. All identified hazards were sampled using the corresponding equipment (*air sampling pump APEX-2 0.5–5.0 L*·*min*^−1^ *Casella UK; explosion-proof pump IFC-2 5.0–30 L*·*min*^−1^*, China*). Several hazards with a simultaneous 8 h time-weighted average exposure concentration (C_TWA_), including NH_3_, VCM, welding fume, manganese, inorganic compounds, CaC_2_ dust, and 1, 2-DCE, were calculated through exposure time and C_STE_ (17–26). Finally, detection results would be evaluated as qualified or disqualified in accordance with the Chinese standard of occupational exposure limits for chemical agents (27).

**Table 1:** Information of identified hazards and detection standards.

**Hazards**	**OEL mg**·**m**^**−3**^		**National** **standards**	**Methods**	**Instruments**
	**MAC/PC-STEL**	**PC-TWA**			
Ammonia (NH_3_)	30	20	GBZ/T160.29-2004	Nanoreagent spectrophotometry	UNIC 2100 spectrophotometer
Chlorine (Cl_2_)	1	—	GBZ/T160.37-2004	Methyl orange spectrophotometry	UNIC 2100 spectrophotometer
Ozone (O_3_)	0.3	—	GBZ/T300.48-2017	Eugenol spectrophotometry	UNIC 2100 spectrophotometer
Hydrochloric acid (HCl)	7.5	—	NIOSH 7907	Volatile acids by Ion Chromatography	Hydrogen flame ionization detector
Sodium hydroxide (NaOH)	2	—	GBZ/T300.22-2017	Flame atomic absorption spectrometry	Flame atomic absorption spectrophotometer
Vinyl chloride monomer (VCM)	—	10	GBZ/T300.78-2017	Thermo desorption gas chromatography	Hydrogen flame ionization detector
Polyvinyl chloride dust (PVC dust)	—	5	GBZ/T 192.1-2007	Membrane filter sampling	Membrane weighting
Welding fume	—	4	GBZ/T 192.1-2007	Membrane filter sampling	Membrane weighting
Calcium carbide dust (CaC_2_ dust)	—	8	GBZ/T 192.1-2007	Membrane filter sampling	Membrane weighting
Manganese and inorganic compounds	—	0.15	GBZ/T300.17-2017	Acid digestion Flame atomic absorption spectrometry	Acetylene-air Flame atomic absorption spectrophotometer
Sulfuretted hydrogen (H_2_S)	10	—	GBZ/T160.33-2004	Silver nitrate colorimetry	Visual colorimetric determination
1, 2-dichloroethane (1, 2-DCE)	15	7	GBZ/T160.45-2007	Solvent absorption gas Chromatography	Hydrogen flame ionization detector

The OEL of calcium carbide dust had not been established in Chinese standard yet so far and it temporarily referred to the OEL of total dust category (PC-TWA = 8 mg·m^−3^).

#### Detection of fresh air requirements and ventilation air changing rate

The fresh air requirements [m^3^/(people·h)] and ventilation air changing rates (t/h) at plants were either detected through the electronic anemometer (ranging between 0.5 and 1 m/s and 1 and 30 m/s, China) or collected from evaluation reports, with specific detection methods lived up to the standard of (28) *GB/T 18204.1-2013 Methods of Hygienic Examination at Public Places Part 1 Physical Factors*, and corresponding Equations (1)–(3) for average wind speed, fresh air requirements, and ventilation air changing rates were displayed as follows:


(1) $$\begin{array}{l} \bar{V}=\frac{\left(V_{1}+V_{2}+.....V_{n}\right)}{n}, \end{array}$$

where $\bar{V}$ represents the average wind speed of a certain vent (m/s), *n* indicates amounts of small, subdivided areas on a certain vent, and *V*_1_ to *V*_*n*_ indicates the average wind speed values detected from subdivided areas in the following equation:


(2) $$Q=\frac{\sum_{\text{i}=\text{1}}^{\text{n}}\left(\text{3600}\times \text{S}\times \bar{V}\right)}{P},$$

where *Q* indicates the fresh air requirements [m^3^/(people·h)], *n* indicates the number of vents at plants, *S* represents the cross-sectional area at a certain vent (m^2^), $\bar{V}$ represents the average wind speed of a certain vent (m/s), and *P* represents the actual maximum number of workers (people):


(3) $$\begin{array}{l} \text{A}=\text{Q}\times \text{P}/\text{V} \end{array}$$

*A* represents ventilation rates (t/h), *Q* indicates the fresh air requirements [m^3^/(people·h)], *P* represents the actual maximum amount of workers (people), and *V* represents the room volume (m^3^).

### Occupational health risk assessment models

The semi-quantitative comprehensive index model, the quantitative model of the International Council on Mining and Metals (ICMM), and the occupational hazards classification model at workplaces (dust and chemical agents) were used to evaluate risk assessment levels in Y and Z before and after improvements on protective measures.

#### The semi-quantitative comprehensive index model

Hazard rank (HR) and exposure rank (ER) were essential components for risk (R), as Equation (7) displayed, and it could be sequentially classified into negligible (R = 1), low (R = 2), medium (R = 3), high (R = 4), and extremely high groups (R = 5). Concretely, HR was assigned certain values with regard to toxicity classification for chemical hazards from the American Conference of Governmental Industrial Hygienists (ACGIH). ER was comprehensively evaluated through Equation (4), in which parameters from *EI*_1_ to *EI*_*n*_, respectively, symbolized vapor pressure/particle size; E/(OEL × *f* ) includes engineering protection measures, first-aid facilities, mode of personal protective tools, emergency rescue measures, occupational health management, weekly usage amount, and weekly contact time. Relevant equations are followed by (4), (5), (6), and (7):


(4) $$\begin{array}{l} \text{ER}\;={\left[EI_{1}\times EI_{2}\times EI_{3}\times EI_{\text{n}}\right]}^{\frac{1}{n}} \end{array}$$

E/OEL represents the ratio between exposure concentration (E) and corresponding occupational exposure limits (OEL). E was calculated through Equation (5) when weekly working hours were mostly equal to 40 h, and the relevant OEL should multiply by a declining factor *f* when daily working hours (H) were longer than 8 h/day. Equations (5) and (6) are written as follows:


(5) $$\begin{array}{l} \text{E}=\text{F}\times \text{D}\times \text{M}/\text{W}, \end{array}$$

where E represents the weekly exposure concentration (mg/m^3^). F refers to the weekly exposure frequency (d/W), and D indicates the average exposure time per day (h/d), M represents the arithmetic weighted mean of exposed concentration (mg·m^−3^), and W means the average weekly working hours, which were limited to 40 h/w:


(6) $$\begin{array}{l} f=\frac{8}{H}\times \frac{\left(24-H\right)}{16} \end{array}$$

(7) $$\begin{array}{l} \text{R}=\sqrt{HR\times ER} \end{array}$$

#### The ICMM quantitative model

Evaluation of the ICMM quantitative model could be calculated through Equation (8), and the risk (R) could be classified into levels of tolerable (<20), potential (20–69), high (70–199), very high (200–399) and intolerable (≥400) groups:


(8) $$\begin{array}{l} R=C\times PrE\times PeE\times U \end{array}$$

Among these, C represents possible consequences for five grades, which are composed of minor illness (C = 1), major illness (C = 7), serious illness (C = 15), major disability (C = 50), and one or more fatalities (C = 100). PrE indicated the possibility of exceeding OEL, which could be classified as 0.5, 1, 3, 6, and 10, as it referred to the extent of the conceivable but very unlikely, only remotely possible, unusual but possible, and intermittently and continuously exceeding. Then, PeE could be classified as 0.5, 1, 2, 3, 6, and 10 according to relevant periods of exposure for rare (once per year), unusual (a few minutes per year), short periods of the month (a few minutes per month), continuous for 1 or 2 h per shift, continuous for 2 or 4 h per shift, and continuous for 8 h per shift. U represented uncertainty assignment for risk rating and exposure assessment, which was allocated to certain (U = 1), uncertain (U = 2), and even quite uncertain (U = 3) (29).

#### Occupational hazards classification method at workplaces

It specialized in the classification of dust and chemical agents with standards of (30) *GBZ/T 229.1-2010 classification of occupational hazards at workplaces, Part 1: occupational exposure to industrial dust*, and (31) *GBZ/T 229.2-2010 classification of occupational hazards at workplaces, Part 2: occupational exposure to chemicals*. Equations (9) and (10) for industrial dust or chemical agents are shown as follows:


(9) $$\begin{array}{l} G=W_{M}\times W_{B}\times W_{L\;} \end{array}$$

(10) $$\begin{array}{l} G=W_{D}\times W_{B}\times W_{L}, \end{array}$$

where *G* corresponds to the risk classification induced by dust and chemical agents, *W*_*M*_ indicates the weight of industrial dust, which was assigned according to the different contents (%) of free silica in the dust; *W*_*D*_represents the weight of chemical agents, graded according to the standard of *GBZ 230–2010 “Classification for Hazards of Occupational Exposure to Toxicants”* (32), *W*_*B*_ represents the weight of E/OEL in chemical agents or industrial dust, and *W*_*L*_ was the weight of workers' physical labor intensity, estimated through the standards of *GBZ/T 189.10–2007, “Measurement of Physical Agents in the Workplace, Part 10: Classification of Physical Workload*” (33) and *GBZ 2.2-2007, “Occupational Exposure Limits for Hazards in the Workplace, Part 2: Physical Hazards”* (34). Ultimately, G would be classified as harmless (G = 0), mildly hazardous (0 <G ≤ 6), moderately hazardous (6 <G ≤ 24), and severely hazardous (G > 24).

### Risk ratio based on risk level conversion

As positions' risk levels under different models were incomparable, they needed to be converted into a kind of homogeneous risk ratio (RR) for quantitative comparisons among different models. Particularly, RR represents the ratio between a certain risk level and the total amount of risk classification. It could be classified into four grades: low risk (0 <RR ≤ 0.25), medium risk (0.25 <RR ≤ 0.5), high risk (0.5 <RR ≤ 0.75), and extremely high risk (0.75 <RR ≤ 1) (35).

### Statistical methods

Epidata 3.0 and SPSS 24.0 were utilized for database establishment and statistical analysis. The Kolmogorov-Smirnov test was conducted for normal distribution judgment, in which data that followed the inclusion criteria were presented by mean ± standard deviation (SD), while abnormally distributed data were alternatively presented by *M* (*P*_25_, *P*_75_). Comparison differences toward abnormal rates were carried out by the *X*^2^ test, and quantitative data were analyzed by the Student's *t*-test or the Mann–Whitney U test. *Spearman's* correlation was used to verify the relative correlation between ambient concentration and ventilation effect data. Multivariate *ANOVA* analysis was used to explore possible independent variables that contributed to multi-dependent variables, and the interactive effect between bilateral variables was done through *LSD*. Logistical linear regression analysis was adopted to analyze different contributions to nervous system symptoms from available variables. Statistical significance for two-tailed *P*-values was defined as α <0.05.

## Results

### Investigation of technological process

Y was a large-scale PVC factory with an annual production of 800,000 tons/year of PVC and 610,000 tons/year of VCM using the technique of acetylene hydrochlorination. Y could be divided into divisions of ore breaking, acetylene generation, chemical synthesis, steam stripping, outward processing, VCM polymerization, refrigeration, product packaging, and other auxiliary ones. By comparison, Z was a VCM manufacturing factory with an annual production of 500,000 tons/year on VCM and of 400,000 tons/year on 1,2-DCE (C_2_H_4_Cl_2_) through the technique of ethylene oxychlorination. It mainly contained sectors such as material storage, splitting decomposition, chemical reactions, oxychlorination, material recycling, field sampling, central control, maintenance, and public engineering. On average, workers in Y and Z worked a 12 h/d shift with 42 h per week. They usually wore anti-poison or anti-dust respirators with earplugs, safety helmets, and uniforms. Reportedly, they also complied with occupational health management disciplines and attended a safety training program held regularly to intensify the awareness of occupational health.

#### Introduction of the acetylene hydrochlorination technique

**Figure 1:**
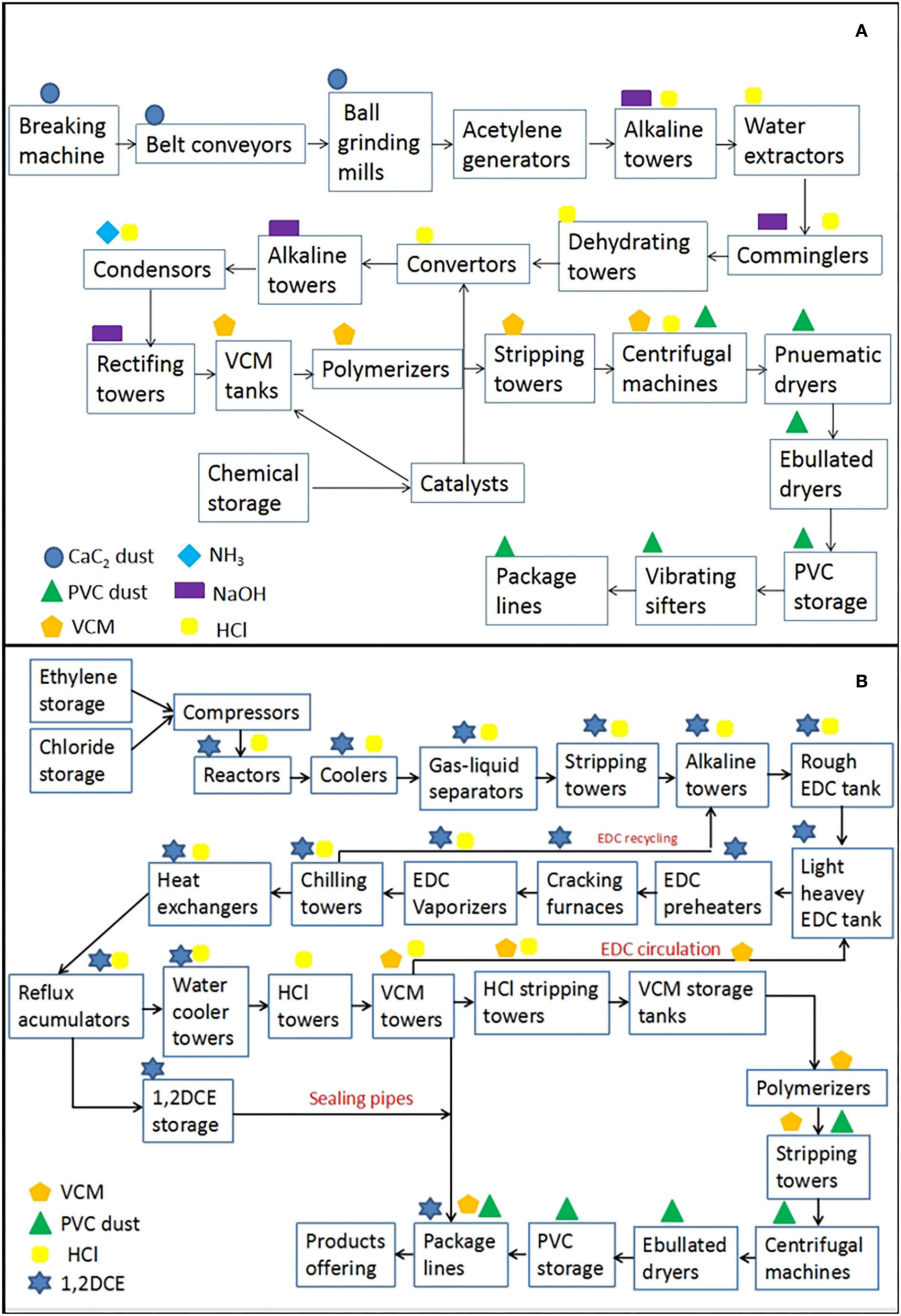
Introduction of technological processes for VCM and PVC production toward the acetylene hydrochlorination technique **(A)** and the ethylene oxychlorination technique **(B)**. In that, the technical process of **(A)** was roughly divided into sectors of ore breaking, acetylene generation, chemical synthesis, steam stripping, outward processing, VCM polymerization, refrigeration, product packaging, and other auxiliary ones. Occupational hazards mainly contained VCM, PVC dust, CaC_2_ dust, NH_3_, HCl (36–38%), NaOH (3.5%), and so on. Process **(B)** were sectors of material storage, splitting decomposition, chemical reaction, oxychlorination, material recycling, field sampling, central control, maintenance, and public engineering. Occupational hazards mainly included VCM, 1, 2-DCE, HCl (36–38%), and PVC dust.

As Figure 1A indicated, the acetylene hydrochlorination technique could be described as a first-order chemical reaction between calcium carbide and water to generate acetylene (C_2_H_2_) and calcium hydroxide Ca (OH)_2_, and then, a second-order chemical reaction was initiated with the precondition of a heated environment between purified acetylene (C_2_H_2_) and hydrogen chloride (HCl) with the acceleration effect of mercury chloride (HgCl_2_) to catalyze vinyl chloride gas (VCM) and then aggregated into polyvinyl chloride (PVC) under high temperature, and products went through the combined lines of hand-packing and automation after centrifugation and desiccation, with all reactions presented in Equations (11) and (12) (36):


(11) $$\begin{array}{l} \text{Ca}{\text{C}}_{2}+{\text{H}}_{2}\text{O}\to {\text{C}}_{2}{\text{H}}_{2}+\text{Ca}{\left(\text{OH}\right)}_{2} \end{array}$$

(12) $$\begin{array}{l} \text{CH}=\text{CH}+\text{HCl}\overset{\text{HgC}{\text{l}}_{2}}{\to }\text{C}{\text{H}}_{2}=\text{CHCl} \end{array}$$

#### Introduction of the ethylene oxychlorination technique

The ethylene oxychlorination technique was operated in three phases, as shown in Figure 1B: (1) the material of chloride (Cl_2_) reacted with ethylene (C_2_H_4_) at a relatively lower temperature environment with the catalysis of ferric trichloride (FeCl_3_) to generate 1, 2-DCE (C_2_H_4_Cl_2_), and then a portion of the qualified 1, 2-DCE would be purified for intermediate products, while the rest would be recycled and steamed out and transmitted into a second step for disintegration. (2) The 1, 2-DCE would be split into VCM gas and HCl at a high reaction temperature inside a sealed cracking furnace. (3) VCM, 1, 2-DCE, and HCl were distinctively isolated through temperature-controlled screening in quench converters, and the purified VCM would be steam-stripped and distributed to the downstream division for polymerization. Chemical reactions are exhibited in Equations (13) and (14) (37):


(13) $$\begin{array}{l} \text{C}{\text{l}}_{2}+{\text{C}}_{2}{\text{H}}_{4}\overset{\text{FeC}{\text{l}}_{3}}{\to }{\text{C}}_{2}{\text{H}}_{4}\text{C}{\text{l}}_{2} \end{array}$$

(14) $$\begin{array}{l} {\text{C}}_{2}{\text{H}}_{4}\text{C}{\text{l}}_{2}\to {\text{C}}_{2}{\text{H}}_{3}\text{Cl}+\text{HCl} \end{array}$$

### Questionnaire analysis

As Table 2 indicated, significant differences in the constituent ratio involved were found on the following parameters: age, length of employment, weekly exposure time, smoking status, alcohol consumption, and sleeping quality between Y and Z before improvement took place in 2020 (*P* < 0.05 or *P* < 0.001). Population distribution by gender between Y and Z was largely homogeneous, as the sex ratio of men to women in Y and Z were 5:1 (341 vs. 67) and 3.7:1 (275 vs. 74), respectively, without significant differences (*X*^2^ = 2.84*, P* > 0.05). Next, they were slightly different in age and length of employment, as ~78.2% of workers in Y were middle-aged men (50.7% were 36–45 years old, and 27.5% were 46–55 years old), while nearly 36.7% of workers in Z were young ones under 35 years. Specifically, statistical differences of age and gender were observed neither between men (44.65 ± 9.40 years) in Y and men (43.65 ± 8.43 years) in Z (*t* = 3.23, *P* > 0.05), nor were they observed between women (44.53 ± 9.21 years) in Y and women (41.17 ± 6.04)] in Z (*t* = 2.27, *P* > 0.05). Occupationally, the percentage toward the length of employment for workers in Y and Z mainly concentrated on a subgroup of 6–20 years, and significant differences were observed between men (18.91 ± 6.67 years) in Y and men (14.46 ± 8.50 years) in Z (*t* = 11.46, *P* < 0.001) and between women (17.2 ± 9.41 years) in Y and women (13.95 ± 4.73 years) in Z (*t* = 9.01, *P* < 0.001). Further, it is worth mentioning that workers under both techniques were under a huge amount of workload with high intensity and density, which might be potentially harmful to their physical health. In addition, it could be noted that the proportion of 54.2% workers in Y were on the 8-h shift and another 33.8% ones were in their 12-h shift pattern respectively, which accounted for a total of 96.6% of laborers in Y working longer than 40 h per week and nearly 73.5% of them sleeping shorter than 6 h per day (56.6% on the 4–6 h and 16.9% on the 0–4-h shift), while the same situation persisted in Z, as shown in Table 2. In addition, it could be noted that the ventilation effect in Y might need to be improved based on the practical experiences or witness as 55.6% of workers argued part of ventilation facilities were temporarily suspended and approximately 19.4% other ones identified fully broken, while 51.9% of workers and 15.5% of others in Z stated the similar statuses.

**Table 2:** Comparison results of questionnaires between Y and Z in 2020.

**Parameters**	**Y (*****n*** = **408)**		**Z (*****n*** = **349)**		***X^2^, P*-value**
**Gender**	**Number**	**%**	**Number**	**%**	
Male	341	83.6	275	78.8	2.84, 0.090
Female	67	16.4	74	21.2	
**Age (years)**
−35	89	21.8	128	36.7	23.65, <0.001^**^
−45	207	50.7	126	36.1	
−55	112	27.5	95	27.2	
**Length of employment (years)**
−5	58	14.2	112	32.1	89.14, <0.001^**^
−20	182	44.6	195	55.9	
−35	168	41.2	42	12.0	
**Working shift system**
8 h day-shift	49	12.0	62	17.8	9.194, 0.010
8 h shift	221	54.2	199	57.0	
12 h night-shift	138	33.8	88	25.2	
**Weekly working time (h)**
−40	14	3.4	21	6.0	2.85, 0.091
−60	394	96.6	328	94.0	
**Weekly exposure time (h)**
−20	96	23.5	48	13.8	10.33, 0.001^*^
−40	312	76.5	301	86.2	
**Smoking status**
Yes	225	55.1	145	41.5	13.92, <0.001^**^
No	183	44.9	204	58.5	
**Alcohol consumption**
Yes	262	64.2	93	26.7	106.60, <0.001^**^
No	146	35.9	256	73.4	
**Sleeping duration (h)**
−4	69	16.9	36	10.3	10.169, 0.006^*^
−6	231	56.7	192	55.0	
−8	108	26.5	121	34.7	
**Sleeping quality**
Good	149	36.5	283	81.1	152.52, <0.001^**^
General	214	52.5	55	15.8	
Bad	45	11.0	11	3.2	
**Individual protective masks**
Always wearing	336	82.3	288	82.5	3.10, 2.212
Sometimes if necessary	59	14.5	42	12.0	
Never	13	3.2	19	5.4	
**Status of ventilation installment**
Normal operation	102	25.0	114	32.7	5.99, 0.051
Temporary suspension	227	55.6	181	51.9	
Fully broken	79	19.4	54	15.5	

* Presented *P* < 0.05 and

** presented *P* < 0.001 as parameters between Y and Z were compared.

### Ambient concentration detection

As Table 3 presented, in 2020, positions in Y of ore breaking (C_TWA_ = 28.4 mg·m^−3^) and acetylene generation (C_TWA_ = 13.5 mg·m^−3^) that were exposed to CaC_2_ dust and steam stripping (C_TWA_ = 11.7 mg·m^−3^), outward processing (C_TWA_ = 15.2 mg·m^−3^), welding maintenance (C_TWA_ = 11.2 mg·m^−3^) that mainly exposed to VCM were disqualified as compared to OEL. No disqualification results were found in Z. Comparatively, those who were disqualified in Y in 2020 declined to qualify in 2021. Correspondingly, comparison results of C_TWA_ toward VCM, PVC dust, CaC_2_ dust, and so on between 2020 and 2021 presented that significant differences were observed in Y (*t* = 2.847, *P* = 0.016, 95% CI = 1.36–10.6) and Z (*t* = 2.40, *P* = 0.030, 95% CI = 0.08–1.27).

**Table 3:** Results of hazards detection before and after improvements in protective measures.

**Positions**	**Cumulative exposure time (h)**	**Hazards**	**2020**			**2021**		
			**C_STE_/C_M_**	**C_TWA_**	**Judgment of results**	**C_STE/_C_M_**	**C_TWA_**	**Judgment of results**
Ore breaking	5	CaC_2_ dust	45.4 (18.1–72.7)	28.4 (11.3–45.4)	Disqualified^a^	6.5 (1.7–11.2)	4.1 (1.1–7.0)	Qualified
Acetylene generation	6	CaC_2_ dust	18.0 (2.2–33.8)	13.5 (1.7–25.3)	Disqualified^a^	6.6 (0.4–6.2)	2.5 (0.3–4.7)	Qualified
Chemical synthesis	3	VCM	<0.9	<0.9	qualified	<0.9	<0.9	Qualified
	1	NaOH	<0.016	—	qualified	<0.016	—	Qualified
Steam stripping	3	VCM	29.2 (4.3–54.1)	11.7 (1.6–20.3)	Disqualified^a^	11.3 (2.5–20.0)	4.5 (1.5–7.5)	Qualified
Outward processing	3	VCM	40.4 (7.5–73.3)	15.2 (2.8–27.5)	Disqualified^a^	6.2 (0.4–12.0)	2.3 (0.2–4.5)	Qualified
VCM polymerization	3	VCM	23.9 (4.0–43.7)	8.9 (1.5–16.4)	Qualified	6.5 (0.2–12.7)	4.9 (0.08–4.8)	Qualified
refrigeration	3	NH_3_	2.9 (2.7–3.0)	1.1 (1.0–1.1)	Qualified	0.3 (0.2–0.4)	0.1 (0.08–0.2)	Qualified
Product packaging	6	PVC dust	3.5 (0.4–6.5)	2.6 (0.3–4.9)	qualified	1.3 (0.2–1.1)	0.3 (0.1–0.4)	Qualified
Welding and repairing	6	VCM	14.9 (1.3–28.5)	11.2 (1.0–21.4)	Disqualified^a^	4.7 (0.3–9.1)	3.5 (0.2–6.8)	Qualified
	3	Welding fume	1.1 (0.2–1.9)	0.8 (0.2–1.4)	Qualified	0.7 (0.2–1.2)	0.6 (0.2–0.9)	Qualified
	3	manganese and inorganic compounds	<0.02	<0.02	Qualified	<0.02	<0.02	Qualified
	3	O_3_	<0.02	—	Qualified	<0.02	—	Qualified
Laboratory testing	5	VCM	3.5(1.1–5.8)	2.2(0.7–3.6)	Qualified	1.5 (0.2–2.8)	1.0 (0.1–1.8)	Qualified
	1	Cl_2_	<0.2	—	Qualified	<0.2	—	Qualified
	1.5	HCl	<0.027	—	Qualified	<0.027	—	Qualified
	1.5	NaOH	<0.016	—	Qualified	<0.016	—	Qualified
Sewage cleaning	5	VCM	<0.9	<0.9	Qualified	<0.9	<0.9	Qualified
	1.5	H_2_S	<0.53	—	Qualified	<0.53	—	Qualified
Material storage	3	VCM	<0.9	<0.9	Qualified	<0.9	<0.9	Qualified
	3	1,2-DCE	<0.56	<0.56	Qualified	<0.56	<0.56	Qualified
	1	HCl	<0.027	—	Qualified	<0.027	—	Qualified
	1	Cl_2_	<0.2	—	Qualified	<0.2	—	Qualified
Splitting decomposition	6	VCM	<0.9	<0.9	Qualified	<0.9	<0.9	Qualified
	3	1,2-DCE	<0.56	<0.56	Qualified	<0.56	<0.56	Qualified
	1	HCl	<0.027	—	Qualified	<0.027	—	Qualified
	1	Cl_2_	<0.2	—	Qualified	<0.2	—	Qualified
Chemical reaction	6	1,2-DCE	2.9 (2.4–3.3)	2.2 (1.8–2.5)	Qualified	1.4 (0.2–2.6)	1.1 (0.2–2.0)	Qualified
	1	Cl_2_	<0.2	—	Qualified	<0.2	—	Qualified
	1	NaOH	<0.016	—	Qualified	<0.016	—	Qualified
Oxychlorination	4	1,2-DCE	1.6 (1.0–2.2)	0.8 (0.5–1.1)	Qualified	1.1 (0.5–1.8)	0.6 (0.3–0.9)	Qualified
	4	VCM	<0.9	<0.9	Qualified	<0.9	<0.9	Qualified
	1	Cl_2_	<0.2	—	Qualified	<0.2	—	Qualified
Material recycling	6	1,2-DCE	6.3 (2.1–10.4)	4.7 (1.6–7.8)	Qualified	4.8 (1.1–8.5)	3.6 (0.8–6.4)	Qualified
	6	VCM	3.3 (0.7–5.9)	2.5 (0.5–4.4)	Qualified	2.4 (0.3–4.5)	1.8 (0.2–3.4)	Qualified
	1.5	HCl	<0.027	—	Qualified	<0.027	—	Qualified
Field sampling	7	1,2-DCE	6.7 (1.1–12.3)	5.9 (1.0–10.8)	Qualified	2.1 (0.6–3.6)	1.6 (0.5–2.7)	Qualified
	7	VCM	1.8 (0.7–2.9)	1.6 (0.6–2.5)	Qualified	1.4 (0.5–2.3)	1.2 (0.4–2.0)	Qualified
Central controlling	6	VCM	<0.9	<0.9	Qualified	<0.9	<0.9	Qualified
Maintenance	6	VCM	2.9 (2.7–3.0)	2.2 (2.0–2.3)	Qualified	1.6 (0.2–3.0)	1.0 (0.1–0.9)	qualified
	6	1,2-DCE	3.1 (0.3–5.9)	2.3 (0.2–4.4)	Qualified	0.6 (0.2–1.0)	0.5 (0.1–0.8)	Qualified
Public engineering	6	VCM	<0.9	<0.9	Qualified	<0.9	<0.9	Qualified
	6	1,2-DCE	<0.56	<0.56	Qualified	<0.56	<0.56	Qualified

a Presented to disqualified results as compared to OEL of certain hazards in manner of C_M_ or C_STE_.

### Fresh air requirement and ventilation air changing rate

Room volumes and vent areas remained the same before and after the improvement. As Table 4 showed, indoor wind speed in Y significantly increased to 15.21 (14.24, 15.89) m/s in 2021 from 6.04 (5.21, 6.40) m/s in 2020 (*Z* = −12.59, *P* < 0.001). Fresh air requirements in Y significantly increased to 30,693.60 (28,602.00, 32,486.40) [m^3^/(people·h)] in 2021 from 10,631.52 (9,216.00, 13,413.60) [m^3^/(people·h)] in 2020 (*Z* = −15.59, *P* < 0.001). Ventilation air changing rates increased to 15.56 (13.30, 16.72) t/h in 2021 from ones of 5.76 (4.66, 6.82) t/h in 2020 (*Z* = −13.77, *P* < 0.001), with statistical differences sequentially. Meanwhile, indicators in Z enhanced to 15.98 ± 0.85 m/s (*Z* = −11.70, *P* < 0.001), 28,925.17 ± 1,317.04 [m^3^/(people·h)] (*Z* = −13.12 *P* < 0.001), and 13.78 ± 0.41 t/h (*Z* = −17.25, *P* < 0.001) in 2021 from 5.39 (4.65, 6.17) m/s, 9,072.00 (8,566.20, 11,315.70) [m^3^/(people·h)] and 4.51 (4.24, 5.29) t/h in 2020 with significant differences.

**Table 4:** Comparison results of wind speed, fresh air requirement and ventilation air changing rate.

**Factories**	**Worksites**	**2020**			**2021**			***Z*, *P*-value**
		**Wind speed^a^** **(m/s)**	**Fresh air requirement^b^ [m^3^/(people·h)]**	**Ventilation air changing rate^c^** **(t/h)**	**Wind speed^d^ (m/s)**	**Fresh air requirement [m^3^/(people·h)]^e^**	**Ventilation air changing rate^f^ (t/h)**	
Y	Crushing plant	6.42	10,631.52	5.76	14.42	23,879.52	12.93	1–4: −12.59, <0.001* 2–5: −15.59, <0.001* 3–6: −13.77, <0.001^*^
	Generating plant	6.40	9,216.00	3.75	22.40	32,256.00	13.14	
	1# Compressor plant	5.21	9,378.00	4.74	15.21	27,378.00	13.85	
	1#Converter plant	5.89	10,602.00	5.36	15.89	28,602.00	14.46	
	2#Material filling plant	4.09	7,362.00	3.38	16.09	28,962.00	13.30	
	1# Polymerization plant	7.54	16,829.28	8.66	13.54	30,221.28	15.56	
	1#Stripping plant	6.32	14,106.24	7.18	15.32	34,194.24	17.40	
	2# Polymerization plant	6.04	13,046.40	6.72	15.04	32,486.40	16.72	
	2#Stripping plant	6.21	13,413.60	6.82	14.21	30,693.60	15.62	
	3#Polymerization plant	5.28	11,404.80	5.87	15.28	33,004.80	16.99	
	3#Stripping plant	4.24	9,158.40	4.66	14.24	30,758.40	15.65	
	Refrigeration plant	6.08	9,849.60	5.03	16.08	26,049.60	13.31	
	Package plant	6.26	10,141.20	5.54	14.26	23,101.20	12.62	
	Maintenance plant	5.91	8,510.40	3.02	25.91	37,310.40	13.26	
	Testing laboratory	7.33	11,874.60	6.17	15.33	24,834.60	12.91	
	Sewage treatment plant	5.39	8,731.80	4.42	15.39	24,931.80	12.61	
Z	Storage plant	6.89	14,882.40	7.69	14.89	32,162.40	16.62	1–4: −11.70, <0.001* 2–5: −13.12, <0.001* 3–6: −17.25, <0.001^*^
	Chlorination plant	4.93	8,874.00	4.51	14.93	26,874.00	13.67	
	Controlling room	4.98	10,756.80	4.29	14.98	32,356.80	12.92	
	Cracking plant	4.46	8,028.00	4.11	14.46	26,028.00	13.32	
		4.79	8,622.00	4.84	14.79	26,622.00	14.94	
	Reacting plant	4.51	8,118.00	4.19	14.51	26,118.00	13.49	
	Repair plant	6.04	13,046.40	4.43	17.04	36,806.40	12.50	
	Storage zone	4.20	9,072.00	4.70	15.20	32,832.00	17.00	

^a−*c*^Referred to wind speed (m/s), fresh air requirement [m^3^/(people·h)], and ventilation air changing rate (t/h) in 2020; ^d−*f*^referred to wind speed (m/s), fresh air requirement [m^3^/(people·h)], and ventilation air changing rate (t/h) in 2021.*P < 0.001 as ^a^compared with ^d^, ^b^compared with ^e^, ^c^compared with ^f^.

### Correlation analysis

As Table 5 demonstrated, ambient concentration (C_STE_ or C_M_) in Y connected to fresh air requirement (*r* = −0.48, *P* = 0.032) and ventilation air changing rate (*r* = −0.49, *P* = 0.029) with a moderate negative correlation in 2020 and converted into a much stronger one in 2021 (*r* = −0.76, *P* = 0.015; *r* = −0.81, *P* = 0.011). In the meantime, ambient concentration in Z did not show much negative correlation with fresh air requirements and ventilation air changing rate in 2020 but revealed a weak correlation in 2021 (*r* = −0.27, *P* = 0.044; *r* = −0.24, *P* = 0.042). It could be assumed that improvements in fresh air requirements and ventilation air changing rates significantly correlated to concentration decline in Y as the majority of positions made activities at indoor plants, while these factors did not seem to be the decisive factor in impacting ambient concentration in Z as its intensive facilities with hazards were placed outdoors, and general ventilation was also involved.

**Table 5:** Correlation analysis among ambient concentration, fresh air requirement, and ventilation air changing rate in Y and Z.

**Factories**	**Factors**	**2020**			**2021**		
		**Ambient concentration**	**Fresh air requirement**	**Ventilation air changing rate**	**Ambient concentration**	**Fresh air requirement**	**Ventilation air changing rate**
Y	Ambient concentration	1.000	**—**	**—**	1.000	**—**	**—**
	Fresh air requirement	−0.48^a*^	1.000	**—**	−0.76^a*^	1.000	**—**
	Ventilation air changing rate	−0.49^b*^	0.84^c*^	1.000	−0.81^b*^	0.87^c*^	1.000
Z	Ambient concentration	1.000	**—**	**—**	1.000	**—**	**—**
	Fresh air requirement	−0.21	1.000	**—**	−0.27	1.000	**—**
	Ventilation air changing rate	−0.23	0.78^c*^	1.000	−0.24	0.84^c*^	1.000

Ambient concentration indicated to C_STE_ or C_M_ of Identified occupational hazards at workplaces in Y and Z. ^a^indicated the comparison of fresh air requirement with ambient concentration; ^b^indicated the comparison of ambient concentration with ventilation air changing rate; ^c^indicated to the comparison of fresh air requirement with ventilation air changing rate. *P < 0.05.

### Occupational health risk assessment

#### Semi-quantitative comprehensive index model

The risk (R) of the semi-quantitative comprehensive index model was determined by HR and ER. In that regard, HR was classified to level 5 as VCM was an IARC group 1 carcinogen (G1), and NaOH, HCl, Cl_2_, H_2_S, and manganese and inorganic compounds were classified into level 4 due to corrosive chemical reagents, poisonous gases of irritation and suffocation, or proved to be mutagenic to humans based on limited animal experiments. HR of NH_3_, 1, 2-DCE, welding fume, PVC dust, and CaC_2_ dust were in level 3 for irritating substances (pH = 8–12), possible human carcinogens (G2B), or hazardous substances to humans or animals with limited evidence. O_3_ would fall into level 2 due to possible irritation threats to the eyes, nose, and throat.

By contrast, ER was calculated through the corresponding assignment of EI. For instance, vapor pressures (EI_1_) of VCM, 1, 2-DCE, NH_3_, HCl, and NaOH were assigned to 5 as they were at 5.5 × 10^7^ Pa (25°C), 1.3 × 10^6^ Pa (20–25°C), 3.8 × 10^4^ Pa (90°C), 3.2 × 10^4^ Pa (20–25°C), 3.9 × 10^4^ Pa, respectively, which exceeded the highest range of vapor pressure (>13,300 Pa), while O_3_, H_2_S, Cl_2_ were assigned to 4 as the standard atmospheric pressure of 101,325 Pa. Next, particle sizes of PVC dust, welding fume, manganese, and inorganic compounds, CaC_2_ dust were assigned to 4, as they were concentrated within a range of 10–50 μm (dry particulate within a range of 10–100 μm). Weekly usage amount (EI_2_) to materials by-products or products such as CaC_2_ dust, VCM, PVC dust, NaOH, HCl, 1, 2-DCE, NH_3_, and Cl_2_ were approximately one hundred thousand tons per year, so their EI would be assigned to five (>1,000 kg or >1,000 L), welding fume, manganese, inorganic compounds, O_3_, and H_2_S would be assigned to one (almost negligible usage amount <1 kg/ <1L). Further, the worker's weekly contact time (EI_3_) to NaOH, HCl, H_2_S, Cl_2_, and H_2_S was assigned to 1 (<8 h), as it was relatively short, as much as 3.5 h, while other hazards were assigned to 2 (≥8 h, <16 h) or to 3(≥16 h, <24 h), as it ranged from 10.5 to 24.5 h. Hazard control measures were, respectively assigned to relevant values in terms of the on-site survey. (5) The ratio of E/(OEL × *f* ) (EI_9_) was assigned to certain values based on C_TWA_ or C_M_.

Comparative results in Y showed that while positions such as steam stripping, outward processing, VCM polymerization, welding, and repairing declined to medium risk in 2021 from high risk in 2020, ore breaking and acetylene generation decreased to low risk in 2021 from medium ones in 2020, other positions like chemical reaction (medium), refrigeration (low), product packaging (low), the laboratory technician (medium), or sewage cleaning (medium) remained unchanged, even though risk levels affected by Cl_2_, HCl, NaOH, and H_2_S were reduced to low risks in 2021. Vertically, it should be noted that significant differences in risk levels were observed at positions in Y (*Z* = 1.62, *P* = 0.011) between 2020 and 2021, while no such alteration was found in Z (*P* > 0.05), as Tables 6, 7 showed.

**Table 6:** Comparison results among three risk assessment models in 2020.

**Positions**	**Hazards**	**Semi-quantitative comprehensive index model**				**ICMM quantitative model**						**Model of occupational hazards classification at workplaces**				
		**HR**	**ER**	**R**	**Rank**	**C**	**PrE**	**PeE**	**U**	**R**	**Rank**	**W_M_/W_D_**	**W_B_**	**W_L_**	**G**	**Rank**
Ore breaking	CaC_2_ dust	3	3	3	Medium	50	10	6	1	3,000	Intolerable	1	3.5	2	7	Moderate harm
Acetylene generation	CaC_2_ dust	3	3	3	Medium	50	10	10	1	5,000	Intolerable	1	1	2	2	Mild harm
Chemical synthesis	VCM	5	2	3	Medium	50	3	6	1	900	Intolerable	8	0	1.5	0	Relatively harmless
	NaOH	4	2	3	Medium	50	0.5	3	1	75	High	4	0	1.5	0	Relatively harmless
Steam stripping	VCM	5	3	4	High	50	10	6	1	3,000	Intolerable	8	1.2	1.5	14	Moderate harm
Outward processing	VCM	5	3	4	High	50	10	6	1	3,000	Intolerable	8	1.5	1.5	18	Moderate harm
VCM polymerization	VCM	5	3	4	High	50	6	6	1	1,800	Intolerable	8	0	1.5	0	Relatively harmless
Refrigeration	NH_3_	3	2	2	Low	15	3	6	1	270	Very high	8	0	1.5	0	Relatively harmless
Product packaging	PVC dust	3	2	2	Low	50	6	10	1	3,000	Intolerable	1	0	1.5	0	Relatively harmless
Welding and repairing	VCM	5	3	4	High	50	6	10	1	3,000	Intolerable	8	1.1	1.5	13	Moderate harm
	Welding fume	3	2	2	Low	50	3	6	1	900	Intolerable	1	0	1.5	0	Relatively harmless
	manganese and inorganic compounds	3	2	2	Low	50	3	6	1	900	Intolerable	8	0	1.5	0	Relatively harmless
	O_3_	3	2	2	Low	15	3	6	1	270	Very high	1	0	1.5	0	Relatively harmless
Laboratory technician	VCM	5	2	3	Medium	50	6	6	1	1,800	Intolerable	8	0	1	0	Relatively harmless
	Cl_2_	4	2	3	Medium	100	0.5	3	1	150	High	8	0	1	0	Relatively harmless
	HCl	4	2	3	Medium	50	1	3	1	150	High	4	0	1	0	Relatively harmless
	NaOH	4	2	3	Medium	50	1	3	1	150	High	4	0	1	0	Relatively harmless
Sewage cleaning	VCM	5	2	3	Medium	50	3	6	1	900	Intolerable	8	0	1.5	0	Relatively harmless
	H_2_S	4	2	3	Medium	100	1	3	1	300	Very high	8	0	1.5	0	Relatively harmless
Material storage	VCM	5	2	3	Medium	50	1	6	1	300	Very high	8	0	1.5	0	Relatively harmless
	1, 2-DCE	3	2	2	Low	50	1	6	1	300	Very high	3	0	1.5	0	Relatively harmless
	HCl	4	2	3	Medium	50	0.5	3	1	75	High	4	0	1.5	0	Relatively harmless
	Cl_2_	4	2	3	Medium	100	0.5	3	1	150	High	8	0	1.5	0	Relatively harmless
Splitting decomposition	VCM	5	2	3	Medium	50	3	10	1	1,500	Intolerable	8	0	1.5	0	Relatively harmless
	1, 2-DCE	3	2	2	Low	50	3	6	1	900	Intolerable	3	0	1.5	0	Relatively harmless
	HCl	4	2	3	Medium	50	0.5	3	1	150	High	4	0	1.5	0	Relatively harmless
	Cl_2_	4	2	3	Medium	100	0.5	3	1	150	High	8	0	1.5	0	Relatively harmless
Chemical reaction	1, 2-DCE	3	2	2	Low	50	3	10	1	1,500	Intolerable	3	0	1.5	0	Relatively harmless
	Cl_2_	4	2	3	Medium	100	0.5	3	1	150	High	8	0	1.5	0	Relatively harmless
	NaOH	4	2	3	Medium	50	0.5	3	1	75	High	4	0	1.5	0	Relatively harmless
Oxychlorination	1, 2-DCE	3	2	2	Low	50	3	6	1	900	Intolerable	3	0	1	0	Relatively harmless
	VCM	5	2	3	Medium	50	3	6	1	900	Intolerable	8	0	1	0	Relatively harmless
	Cl_2_	4	2	3	Medium	100	0.5	3	1	150	High	8	0	1	0	Relatively harmless
Material Recycling	1, 2-DCE	3	2	2	Low	50	6	10	1	3,000	Intolerable	3	0	1.5	0	Relatively harmless
	VCM	5	2	3	Medium	50	3	10	1	1,500	Intolerable	8	0	1.5	0	Relatively harmless
	HCl	4	2	3	Medium	50	0.5	3	1	75	High	4	0	1.5	0	Relatively harmless
Field sampling	1, 2-DCE	3	2	2	Low	50	6	10	1	3,000	Intolerable	3	0	1.5	0	Relatively harmless
	VCM	5	2	3	Medium	50	3	10	1	1,500	Intolerable	8	0	1.5	0	Relatively harmless
Central controlling	VCM	5	2	3	Medium	50	3	10	1	1,500	Intolerable	8	0	1	0	Relatively harmless
Maintenance	VCM	5	2	3	Medium	50	6	10	1	3,000	Intolerable	8	0	1.5	0	Relatively harmless
	1, 2-DCE	3	2	2	Low	50	6	10	1	3,000	Intolerable	3	0	1.5	0	Relatively harmless
Public engineering	VCM	5	2	3	Medium	50	3	10	1	1,500	Intolerable	8	0	1.5	0	Relatively harmless
	1, 2-DCE	3	2	2	Low	50	3	10	1	1,500	Intolerable	3	0	1.5	0	Relatively harmless
RR [median (range)]^*^		0.6 (0.4–0.8)^a^				0.9 (0.6–1)^b^						0.3 (0.25–0.75)^c^				

The RR [median (range) ] indicated the certain risk level and the total amount of risk classification with the classification of 4 grades, as low risk (0 <RR ≤ 0.25), medium risk (0.25 <RR ≤ 0.5), high risk (0.5 <RR ≤ 0.75), and extremely high risk (0.75 <RR ≤ 1), ^a^presented to 0.6 (0.4–0.8), ^b^presented to 0.9 (0.6–1), ^c^presented to 0.3 (0.25–0.75), *P < 0.001.

**Table 7:** Comparison results among three risk assessment models in 2021.

**Positions**	**Hazards**	**Semi-quantitative comprehensive index model**				**ICMM quantitative model**						**Model of occupational hazards classification at workplaces**				
		**HR**	**ER**	**R**	**Rank**	**C**	**PrE**	**PeE**	**U**	**R**	**Rank**	**W_M_/W_D_**	**W_B_**	**W_L_**	**G**	**Rank**
Ore breaking	CaC_2_ dust	3	2	2	Low	50	6	6	1	1,800	Intolerable	1	0	2	0	Relatively harmless
Acetylene generation	CaC_2_ dust	3	2	2	Low	50	6	10	1	3,000	Intolerable	1	0	2	0	Relatively harmless
Chemical synthesis	VCM	5	2	3	Medium	50	3	6	1	900	Intolerable	8	0	1.5	0	Relatively harmless
	NaOH	4	1	2	Low	50	0.5	3	1	75	High	4	0	1.5	0	Relatively harmless
Steam stripping	VCM	5	2	3	Medium	50	6	6	1	1,800	Intolerable	8	0	1.5	0	Relatively harmless
Outward processing	VCM	5	2	3	Medium	50	6	6	1	1,800	Intolerable	8	0	1.5	0	Relatively harmless
VCM polymerization	VCM	5	2	3	Medium	50	3	6	1	900	Intolerable	8	0	1.5	0	Relatively harmless
Refrigeration	NH_3_	3	1	2	Low	15	3	6	1	270	Very high	8	0	1.5	0	Relatively harmless
Product packaging	PVC dust	3	2	2	Low	50	1	10	1	500	Intolerable	1	0	1.5	0	Relatively harmless
Welding and repairing	VCM	5	2	3	Medium	50	3	10	1	1,500	Intolerable	8	0	1.5	0	Relatively harmless
	Welding fume	3	1	2	Low	50	3	6	1	900	Intolerable	1	0	1.5	0	Relatively harmless
	manganese and inorganic compounds	3	1	2	Low	50	3	6	1	900	Intolerable	8	0	1.5	0	Relatively harmless
	O_3_	3	1	2	Low	15	3	6	1	270	Very high	1	0	1.5	0	Relatively harmless
Laboratory technician	VCM	5	2	3	Medium	50	3	6	1	900	Intolerable	8	0	1	0	Relatively harmless
	Cl_2_	4	1	2	Low	100	0.5	3	1	150	High	8	0	1	0	Relatively harmless
	HCl	4	1	2	Low	50	1	3	1	150	High	4	0	1	0	Relatively harmless
	NaOH	4	1	2	Low	50	1	3	1	150	High	4	0	1	0	Relatively harmless
Sewage cleaning	VCM	5	2	3	Medium	50	3	6	1	900	Intolerable	8	0	1.5	0	Relatively harmless
	H_2_S	4	1	2	Low	100	1	3	1	300	Very high	8	0	1.5	0	Relatively harmless
Material storage	VCM	5	2	3	Medium	50	1	6	1	300	Very high	8	0	1.5	0	Relatively harmless
	1,2-DCE	3	2	2	Low	50	1	6	1	300	Very high	3	0	1.5	0	Relatively harmless
	HCl	4	1	2	Low	50	0.5	3	1	75	High	4	0	1.5	0	Relatively harmless
	Cl_2_	4	1	2	Low	100	0.5	3	1	150	High	8	0	1.5	0	Relatively harmless
Splitting decomposition	VCM	5	2	3	Medium	50	3	10	1	1,500	Intolerable	8	0	1.5	0	Relatively harmless
	1,2-DCE	3	2	2	Low	50	3	6	1	900	Intolerable	3	0	1.5	0	Relatively harmless
	HCl	4	1	2	Low	50	1	3	1	150	High	4	0	1.5	0	Relatively harmless
	Cl_2_	4	1	2	Low	100	0.5	3	1	150	High	8	0	1.5	0	Relatively harmless
Chemical reaction	1,2-DCE	3	2	2	Low	50	3	10	1	1,500	Intolerable	3	0	1.5	0	Relatively harmless
	Cl_2_	4	1	2	Low	100	0.5	3	1	150	High	8	0	1.5	0	Relatively harmless
	NaOH	4	1	2	Low	50	0.5	3	1	75	High	4	0	1.5	0	Relatively harmless
Oxychlorination	1,2-DCE	3	2	2	Low	50	1	6	1	300	Very high	3	0	1	0	Relatively harmless
	VCM	5	2	3	Medium	50	1	6	1	300	Very high	8	0	1	0	Relatively harmless
	Cl_2_	4	1	2	Low	100	0.5	3	1	150	High	8	0	1	0	Relatively harmless
Material recycling	1,2-DCE	3	2	2	Low	50	3	10	1	1,500	Intolerable	3	0	1.5	0	Relatively harmless
	VCM	5	2	3	Medium	50	3	10	1	1,500	Intolerable	8	0	1.5	0	Relatively harmless
	HCl	4	1	2	Low	50	0.5	3	1	75	High	4	0	1.5	0	Relatively harmless
Field sampling	1,2-DCE	3	2	2	Low	50	3	10	1	1,500	Intolerable	3	0	1.5	0	Relatively harmless
	VCM	5	2	3	Medium	50	3	10	1	1,500	Intolerable	8	0	1.5	0	Relatively harmless
Central controlling	VCM	5	2	3	Medium	50	3	10	1	1,500	Intolerable	8	0	1	0	Relatively harmless
Maintenance	VCM	5	2	3	Medium	50	3	10	1	1,500	Intolerable	8	0	1.5	0	Relatively harmless
	1,2-DCE	3	2	2	Low	50	3	10	1	1,500	Intolerable	3	0	1.5	0	Relatively harmless
Public engineering	VCM	5	2	3	Medium	50	3	10	1	1,500	Intolerable	8	0	1.5	0	Relatively harmless
	1,2-DCE	3	2	2	Low	50	3	10	1	1,500	Intolerable	3	0	1.5	0	Relatively harmless
RR [median (range)]^*^		0.5 (0.4–0.6)^a^				0.9 (0.6–1)^b^						0.25^c^				

The RR [median (range)] indicated the certain risk level and the total amount of risk classification with the classification of 4 grades, as low risk (0 <RR ≤ 0.25), medium risk (0.25 <RR ≤ 0.5), high risk (0.5 <RR ≤ 0.75), and extremely high risk (0.75 <RR ≤ 1), ^a^presented to 0.5 (0.4–0.6), ^b^presented to 0.9 (0.6–1), ^c^presented to 0.25, *P < 0.001.

#### Implementation of the ICMM model

Hazard consequence (C) was based on the severity of harm or damage that occurred at workplaces. In this case, C caused by CaC_2_ dust, PVC dust, welding fume, VCM, HCl, NaOH, 1, 2-DCE, and manganese, and inorganic compounds would cause major disabilities (C = 50), NH_3_ and O_3_ might cause serious illness or be absent for longer than 14 days (C = 15), and Cl_2_ and H_2_S were hazardous gases that would cause one or more fatalities (C = 100) even at a minimum concentration. U was determined to be certain (U = 1) throughout the hazards exposed by workers from Y and Z, before and after improvement.

Furthermore, PrE of HCl, NaOH, and Cl_2_ was assigned to the grade “conceivable” but “very unlikely” or “only remotely possible” (PrE = 0.5 or 1) as hazards like these were auxiliary materials in catalysis or neutralization reactions or were frequently used in a laboratory test. Meanwhile, PrE of CaC_2_ dust and VCM would be assigned to “continuously exceeding” (PrE = 10) as C_TWA_ had exceeded the relevant OEL, and PrE of other hazards would be assigned to “unusual but possible or intermittently” (PrE = 3 or 6). PeE of different hazards would be assigned in terms of the exposure period.

The only risk level for oxychlorination changed from an intolerable one in 2020 to a very high one in 2020, while other positions remained unchanged at very high or intolerable risks, respectively. In addition, positions would be at high or very high risk when exposed to strong acids, alkalis, or highly toxic substances, such as Cl_2_, HCl, NaOH, NH_3_, and H_2_S concurrently or any of them alternatively. No significant difference in risk levels among positions in Y or Z was found before and after improvement (*P* > 0.05) using the ICMM model, as shown in Tables 6, 7.

#### Classification of occupational hazards at workplaces

According to a previous survey, the crystalline free silica content (M%) of CaC_2_ dust, PVC dust, and welding fume were estimated to be lower than 10%, as they did not contain many silicates; thus, their *W*_*M*_would be assigned to level 1(M <10%). As regards chemical agents, the hazardous toxicant index (THI) of O_3_ was at mild harm (THI <35) in terms of the calculation equation in GBZ 230-2010; therefore, the *W*_*D*_would also be assigned to level 1 (mild harm). Then Cl_2_, NH_3_, H_2_S, VCM, manganese, and inorganic compounds were all hazards on the *Catalogue of Highly Toxic Substances (2003)* (38), *and* their *W*_*D*_would be assigned to 8 (extreme harm); HCl and NaOH would be assigned to 4 (severe harm), as their THI was in the range of severe harm (50 ≤ THI <65). In addition, *W*_*B*_and *W*_*L*_were, respectively, assigned according to CM or CTWA and manual labor intensity. Positions included ore breaking, acetylene generation, steam stripping, outward processing, welding, and repairing, which were adjusted to relatively harmless in 2021 from mild or moderate harm in 2020. No significant difference in risk classification for positions throughout Y or Z was found between 2020 and 2021 (*P* > 0.05), as Tables 6, 7 show.

#### Comparison results of different models

**Figure 2:**
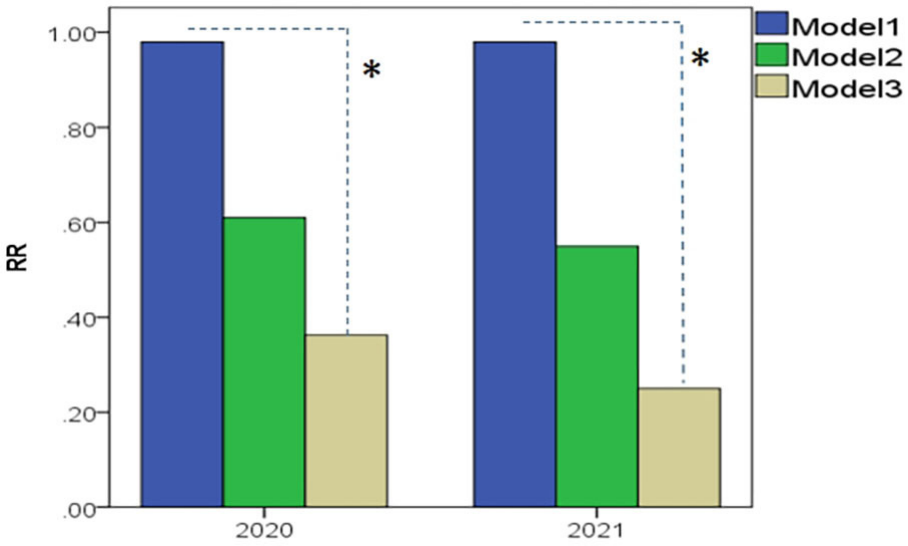
Comparison results of RR among Model 1–3, as they, respectively, represented to ICMM quantitative model, the semi-quantitative comprehensive index model, and the model of occupational hazards classification at workplaces (industrial dust and chemical agents). Model 1: The ICMM quantitative model achieved the highest RR of 0.9 (0.6–1.0) in both 2020 and in 2021; Model 2: RR toward the semi-quantitative comprehensive index model went to 0.6 (0.4–0.8) in 2020 and 0.5 (0.4–0.6) in 2021, respectively; Model 3: the model of occupational hazards classification at workplaces left to the lowest of 0.3 (0.25–0.75) and 0.25. *P < 0.001 as significant differences of RR among models were compared.

The ICMM quantitative model achieved the highest RR of 0.9 (0.6–1.0) in both 2020 and 2021, followed by the semi-quantitative comprehensive index model of 0.6 (0.4–0.8) and 0.5 (0.4–0.6); subsequently, the model of occupational hazards classification at workplaces was at the lowest at 0.3 (0.25–0.75) and 0.25. Significant differences in RR among models in 2020 (*Z* = 19.21, *P* < 0.001) and 2021 (*Z* = 16.01, *P* < 0.001) were observed, respectively. It could be observed that the risk levels using the ICMM model could be frequently elevated to very high or intolerable levels, leading to an overestimated evaluation that would exaggerate the real risk points (39), as indicated in Figure 2.

### Analysis of physical examination results

#### Abnormal rate analysis for liver ultrasound

**Figure 3:**
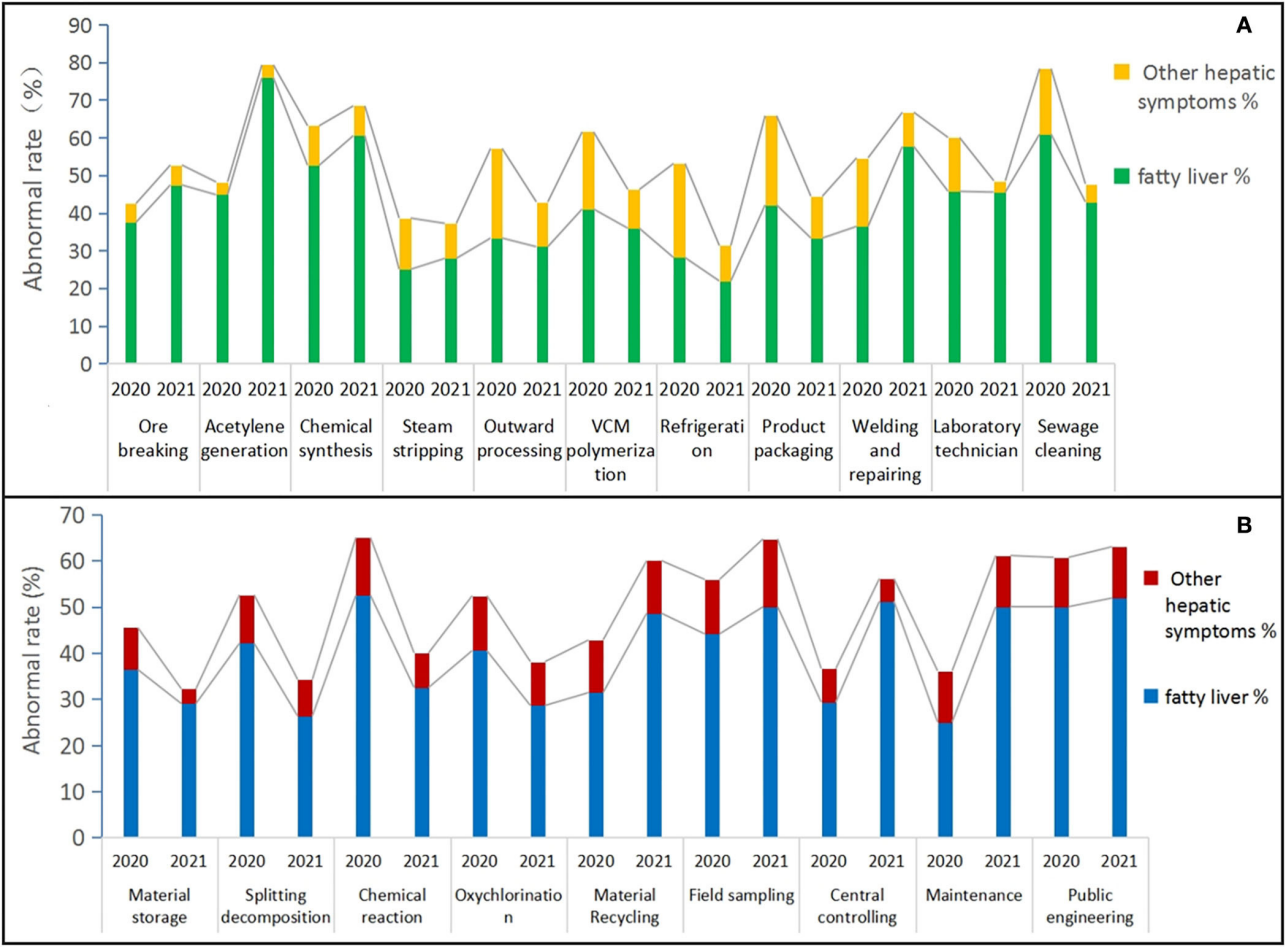
Abnormal rates of alteration toward fatty liver and the other hepatic symptoms among all positions throughout Y and Z between 2020 and 2021 were visualized in histograms **(A,B)**. That chart **(A)** described the distribution of abnormal rates of fatty liver (green) and the other hepatic symptoms (yellow) for 11 positions in Y; Chart **(B)** presented the distribution of abnormal rates of fatty liver (blue) and the other hepatic symptoms (red) for nine positions in Z. Abnormal rates (%) for each position were, respectively, calculated through dividing the abnormal person-time of fatty liver or other hepatic symptoms to the total number of each relevant position.

Given that risk assessment levels toward several positions in Y and Z were found to be different before and after improvements in protective measures from 2020 to 2021. How this effect could be related to physical health status in terms of liver function remains unknown yet. To discover possible alterations toward fatty liver and other hepatic symptoms in liver ultrasound results between 2020 and 2021, abnormal rates (%) were visualized in Figures 3A,B, and data were compared in Tables 8–10. Primarily, it should be noted that the majority of positions in Y and Z showed alterations toward abnormal rates to a different extent. However, significant differences containing fatty liver and other hepatic symptoms were only found in Y (*X*^2^ = 10.19, *P* < 0.001) between 2020 and 2021, and no such changes were ever discovered under a bilateral interaction for year and position categories (*P* > 0.05).

**Table 8:** Results of multiple linear regression analysis for physical exam data in Y in 2020 and 2021.

**Liver ultrasound**	**Factors**	**2020 (*****n*** = **393)**						**2021 (*****n*** = **384)**					
		***B***	***SE***	***WaldX* ^2^**	***P***	***Exp(B)***	***95% CI***	***B***	***SE***	***WaldX* ^2^**	***P***	***Exp(B)***	**95% CI**
Fatty liver	Male	1.116	0.399	7.823	0.005^*^	3.052	1.396–6.669	1.274	0.253	25.404	0.000^**^	3.574	2.178–5.568
	Female	0.123	0.054	0.839	0.360	1.131	0.390–3.281	0.470	0.072	0.468	0.206	1.600	1.142–2.242
	Age	0.056	0.017	0.974	0.201	1.058	1.023–1.093	0.004	0.013	0.073	0.787	1.004	0.978–1.030
	ALT	0.147	0.050	8.630	0.003^*^	1.159	1.050–1.278	−0.005	0.016	0.113	0.737	0.995	0.964–1.027
	AST	−0.130	0.054	5.728	0.017^*^	0.878	0.790–0.977	0.007	0.009	0.599	0.439	1.007	0.989–1.025
	GGT	0.030	0.008	4.495	0.021^*^	1.010	1.013–1.047	0.000	0.008	0.001	0.975	1.000	0.984–1.016
	ALP	0.011	0.007	2.407	0.121	1.011	0.997–1.025	−0.018	0.021	0.699	0.403	0.982	0.942–1.024
	TBIL	−0.012	0.021	0.329	0.566	0.988	0.947–1.030	0.174	0.234	4.100	0.143	1.206	1.015–2.541
	TG	0.590	0.286	4.253	0.009^*^	1.803	1.030–3.159	0.546	0.328	4.223	0.016^*^	1.216	0.529–1.912
	TC	0.440	0.160	3.761	0.018^*^	1.250	0.840–1.574	0.340	0.127	2.362	0.021^*^	1.244	0.921–1.631
Other hepatic symptoms	Male	1.801	0.673	7.169	0.007^*^	6.055	1.620–12.627	1.116	0.009	0.248	0.004^*^	3.276	0.987–3.026
	Female	0.084	0.588	0.416	0.519	1.373	0.524–3.596	−0.005	0.024	0.049	0.825	0.995	0.950–1.042
	Age	0.029	0.022	2.530	0.346	1.127	1.079–1.177	0.012	0.020	0.362	0.548	1.012	0.973–1.053
	ALT	0.172	0.053	10.368	0.001^*^	1.187	1.070–1.318	0.024	0.074	5.548	0.119	3.052	1.206–7.724
	AST	−0.159	0.063	6.359	0.012^*^	0.853	0.754–0.965	0.019	0.057	3.839	0.136	1.521	0.620–3.725
	GGT	0.021	0.009	5.476	0.019^*^	1.022	1.003–1.040	0.002	0.015	0.010	0.920	1.002	0.972–1.032
	ALP	−0.002	0.012	0.036	0.849	0.998	0.982–1.015	0.003	0.018	0.014	0.906	1.010	0.968–1.037
	TBIL	0.002	0.027	0.006	0.940	1.002	0.951–1.055	0.005	0.010	0.247	0.620	1.005	0.985–1.026
	TG	0.177	0.347	0.259	0.611	1.193	0.604–2.356	0.222	0.409	0.295	0.587	1.249	0.561–2.781
	TC	−0.024	0.201	0.015	0.904	0.976	0.658–1.448	0.134	0.202	0.345	0.198	1.202	0.918–2.142

* Presented to *P* < 0.05,

** presented to *P* < 0.001.

**Table 9:** Results of multiple linear regression analysis for physical exam data in Z in 2020 and 2021.

**Liver ultrasound**	**Factors**	**2020 (*****n*** = **327)**						**2021 (*****n*** = **324)**					
		***B***	***SE***	***WaldX* ^2^**	***P***	***Exp(B)***	***95% CI***	***B***	***SE***	***Wald X^2^***	***P***	***Exp (B)***	**95% CI**
Fatty liver	Male	−0.290	0.356	5.662	0.006^*^	1.248	0.372–1.505	−0.287	0.417	4.474	0.004^*^	1.570	0.331–1.699
	Female	−0.049	0.125	0.489	0.312	0.637	0.442–1.291	−0.026	0.043	0.396	0.412	0.787	0.641–1.184
	Age	−0.055	0.019	2.230	0.212	0.937	0.902–0.973	−0.041	0.018	1.081	0.124	0.960	0.927–0.995
	ALT	0.013	0.028	0.221	0.638	1.013	0.959–1.071	−0.028	0.008	3.979	0.064	0.962	0.958–0.987
	AST	0.006	0.039	0.020	0.886	1.006	0.931–1.086	0.019	0.012	2.589	0.108	1.020	0.996–1.044
	GGT	−0.002	0.008	0.038	0.846	0.998	0.983–1.014	−0.012	0.008	2.244	0.134	0.988	0.972–1.004
	ALP	0.004	0.007	0.336	0.562	1.004	0.991–1.018	0.011	0.007	2.458	0.117	1.011	0.997–1.026
	TBIL	0.010	0.035	0.082	0.774	1.010	0.944–1.081	0.012	0.035	0.123	0.725	1.012	0.946–1.083
	TG	0.651	0.179	9.415	0.002^*^	1.734	1.220–2.465	0.462	0.174	7.006	0.008^*^	1.587	1.127–2.234
	TC	0.512	0.267	3.605	0.035^*^	1.336	0.891–1.715	0.382	0.168	6.826	0.033^*^	1.526	0.954–1.843
Other hepatic symptoms	Male	−0.020	0.392	4.372	0.007^*^	1.440	0.204–1.950	−0.066	0.500	4.017	0.006^*^	1.937	0.352–2.495
	Female	−0.019	0.028	0.429	0.310	0.882	0.735–1.008	−0.022	0.019	0.513	0.329	0.871	0.632–1.278
	Age	−0.033	0.023	2.093	0.148	0.967	0.925–1.012	−0.010	0.021	0.220	0.639	0.990	0.950–1.032
	ALT	−0.049	0.041	4.206	0.025^*^	1.012	0.906–1.063	−0.018	0.009	4.237	0.040^*^	1.084	0.966–1.199
	AST	0.032	0.056	3.329	0.036^*^	1.033	0.925–1.153	0.036	0.014	4.165	0.025^*^	1.006	0.978–1.034
	GGT	0.000	0.010	0.000	0.983	1.000	0.981–1.019	−0.015	0.010	2.298	0.130	0.985	0.967–1.004
	ALP	−0.009	0.008	1.125	0.289	0.991	0.975–1.008	0.006	0.008	0.520	0.471	1.006	0.990–1.022
	TBIL	0.018	0.040	0.209	0.648	1.018	0.941–1.012	0.018	0.040	0.199	0.656	1.018	0.941–1.102
	TG	0.369	0.200	3.415	0.065	1.447	0.978–2.141	0.297	0.195	2.323	0.127	1.345	0.919–1.970
	TC	0.290	0.193	2.263	0.133	1.336	0.916–1.949	0.358	0.191	3.501	0.061	1.430	0.983–2.080

* Presented to *P* < 0.05.

**Table 10:** Results of liver ultrasound analysis in Y and Z between 2020 and 2021.

**Factories**	**Positions**	**2020**					**2021**					***X^2^*, *P-*value**
		***n***	**Fatty liver**		**Other hepatic symptoms**		***n***	**Fatty liver**		**Other hepatic symptoms**		
			**Abnormal^a^**	**%**	**Abnormal^b^**	**%**		**Abnormal^c^**	**%**	**Abnormal^d^**	**%**	
Y 2020 (*n* = 393); 2021 (*n* = 384)	Ore breaking	40	15	37.5	2	5.0	38	18	47.4	2	5.3	0.03, 0.86
	Acetylene generation	29	13	44.8	1	3.4	29	22	75.9	1	3.4	0.13, 0.72
	Chemical synthesis	38	20	52.6	4	10.5	38	23	60.5	3	7.9	0.27, 0.60
	Steam stripping	44	11	25.0	6	13.6	43	12	27.9	4	9.3	0.41, 0.52
	Outward processing	42	14	33.3	10	23.8	42	13	31.0	5	11.9	0.86, 0.35
	VCM polymerization	39	16	41.0	8	20.5	39	14	35.9	4	10.3	0.62, 0.43
	Refrigeration	32	9	28.1	8	25.0	32	7	21.9	3	9.4	0.76, 0.38
	Product packaging	38	16	42.1	9	23.7	36	12	33.3	4	11.1	0.55, 0.46
	Welding and repairing	33	12	36.4	6	18.2	33	19	57.6	3	9.1	2.20, 0.13
	Laboratory technician	35	16	45.7	5	14.3	33	15	45.5	1	3.0	2.06, 0.15
	Sewage cleaning	23	14	60.9	4	17.4	21	9	42.9	1	4.8	0.66, 0.42
	Total	393	156	39.7	64	16.3	384	164	42.7	31	8.1	10.19, <0.001^*^
Z 2020 (*n* = 327); 2021 (*n* = 324)	Material storage	33	12	36.4	3	9.1	31	9	29.0	1	3.2	0.45, 0.50
	Splitting decomposition	38	16	42.1	4	10.5	38	10	26.3	3	7.9	0.05, 0.83
	Chemical reaction	40	21	52.5	5	12.5	40	14	32.5	3	7.5	0.02, 0.90
	Oxychlorination	42	17	40.5	5	11.9	42	12	28.6	4	9.5	0.03, 0.87
	Material Recycling	35	11	31.4	4	11.4	35	16	48.6	4	11.4	0.22, 0.64
	Field sampling	34	15	44.1	4	11.8	34	17	50.0	5	14.7	0.02, 0.90
	Central controlling	41	12	29.3	3	7.3	41	21	51.2	2	4.9	1.02, 0.31
	Maintenance	36	10	25.0	4	11.1	36	18	50.0	4	11.1	0.73, 0.49
	Public engineering	28	13	50.0	3	10.7	27	14	51.9	3	11.1	0.01, 0.94
	Total	327	127	38.8	35	10.7	324	131	40.4	29	9.0	0.61, 0.43

* Referred to *P* < 0.001 as compared to abnormal rates of fatty liver and other hepatic symptoms between 2020 and 2021,

a indicated the abnormal number of fatty liver among positions in 2020,

b indicated the abnormal number of other hepatic symptoms among positions in 2020;

c indicated to the abnormal number of fatty liver among positions in 2021, and

d indicated to the abnormal number of other hepatic symptoms among positions in 2021.

And then, in Y, positions referring to increased abnormal rates of fatty liver from 2020 to 2021 touched on acetylene generation (31.1%), welding and repairing (21.2%), or breaking (9.9%), chemical synthesis (7.9%), and steam stripping (2.9%), while sewage cleaning (18.0%), product packaging (7.9%), refrigeration (6.2%), VCM polymerization (5.1%), and outward processing (2.3%) exhibited a decreased tendency, and the laboratory technician (a 0.2% decrease) remained roughly unchanged. With regard to other hepatic symptoms, abnormal rates of most positions in Y exhibited a declining trend except ore breaking (a 0.3% increase) and acetylene generation (0.0%), as Figure 3A presented.

By contrast, in Z, positions of maintenance (25.0%), central control (21.9%), material recycling (17.2%), field sampling (5.9%), and public engineering (1.9%) maintained an increasing trend in abnormal rates toward the fatty liver, while chemical reaction (20.0%), splitting decomposition (15.8%), oxychlorination (11.9%), as well as material storage (7.4%), were in an opposite orientation. As for other hepatic symptoms, material storage (5.9%), a chemical reaction (5.0%), splitting decomposition (2.6%), oxychlorination (2.4%), and central control (2.3%) revealed a slightly dropped tendency, while field sampling presented a slight increase of 2.9%, while others like material recycling (0.0%), maintenance (0.0%), and public engineering (0.4%) kept unchanged, as Figure 3B shows.

#### Analysis of multiple linear regression

This part is intended to evaluate independent variables that might contribute to fatty liver and other hepatic symptoms in both 2020 and 2021 by using multiple linear regression analysis. In Tables 8–10, the variable for males made a straight contribution to fatty liver and other hepatic symptoms in Y and Z for 2 years. Concretely, it played a role in the fatty liver [(*Exp(B)* = 3.052, 95% CI = 1.396–6.669, *P* = 0.005) in 2020, (*Exp(B)* = 3.574, 95%CI = 2.718–5.568, *P* < 0.001) in 2021] and to other hepatic symptoms [(*Exp(B)* = 6.055, 95% CI = 1.620–12.627, *P* = 0.007) in 2020, (*Exp(B)* = 3.276, 95% CI = 0.987–5.026, *P* = 0.004) in 2021] in Y as compared to women; in Z, a similar effect to the fatty liver [(*Exp(B)* = 1.248, 95% CI = 0.372–1.505, *P* = 0.006) in 2020, (*Exp(B)* = 1.570, 95% CI = 0.331–1.699, *P* = 0.004) in 2021] and other hepatic symptoms [(*Exp(B)* = 1.440, 95% CI = 0.204–1.950, *P* = 0.007) in 2020 (*Exp(B)* = 1.937, 95% CI = 0.352–2.495, *P* = 0.006) in 2021] was observed as compared to women.

In Y, variables such as ALT, AST, GGT, TG, and TC were influential factors that contributed to fatty liver in 2020, and in left-handed males, TG and TC had a similar effect in 2021. In the meantime, variables such as ALT, AST, and GGT contributed to other hepatic symptoms in 2020, and no such effect was spotted in 2021 anymore. In Z, only the variables TG and TC contributed to the fatty liver, while ALT and AST contributed to other hepatic symptoms, and these indicators lasted through 2020 and 2021. Next, not surprisingly, variables TG and TC were only found to contribute to fatty liver in Y and Z in both 2020 and 2021, and no such effect was found on other hepatic symptoms. In particular, variables such as ALT, AST, and GGT in Y were observed to contribute to the fatty liver [ALT (*Exp(B)* = 1.159, 95% CI = 1.050–1.278, *P* = 0.003); AST (*Exp(B)* = 0.878, 95% CI = 0.790–0.977, *P* = 0.017); GGT (*Exp(B)* = 1.010, 95% CI = 1.013–1.047, *P* = 0.021)] and to other hepatic symptoms [ALT (*Exp(B)* = 1.187, *95%CI* = 1.070-1.318, *P* = 0.001); AST (*Exp(B)* = 1.153, 95% CI = 0.754–1.965, *P* = 0.012); GGT (*Exp(B)* = 1.022, 95% CI = 1.003–1.040, *P* = 0.019)] in 2020. In contrast, ALT and AST played an important role in Z only for other hepatic symptoms in both 2020 [ALT (*Exp(B)* = 1.012, 95% CI = 0.906–1.063, *P* = 0.025); AST(*Exp(B)* = 1.033, 95% CI = 0.925–1.153, *P* = 0.036)] and 2021 [ALT(*Exp(B)* = 1.084, 95% CI = 0.966–1.199, *P* = 0.040); AST (*Exp(B)* = 1.006, 95% CI = 0.978–1.034, *P* = 0.025)].

#### Analysis of liver function indicators

**Figure 4:**
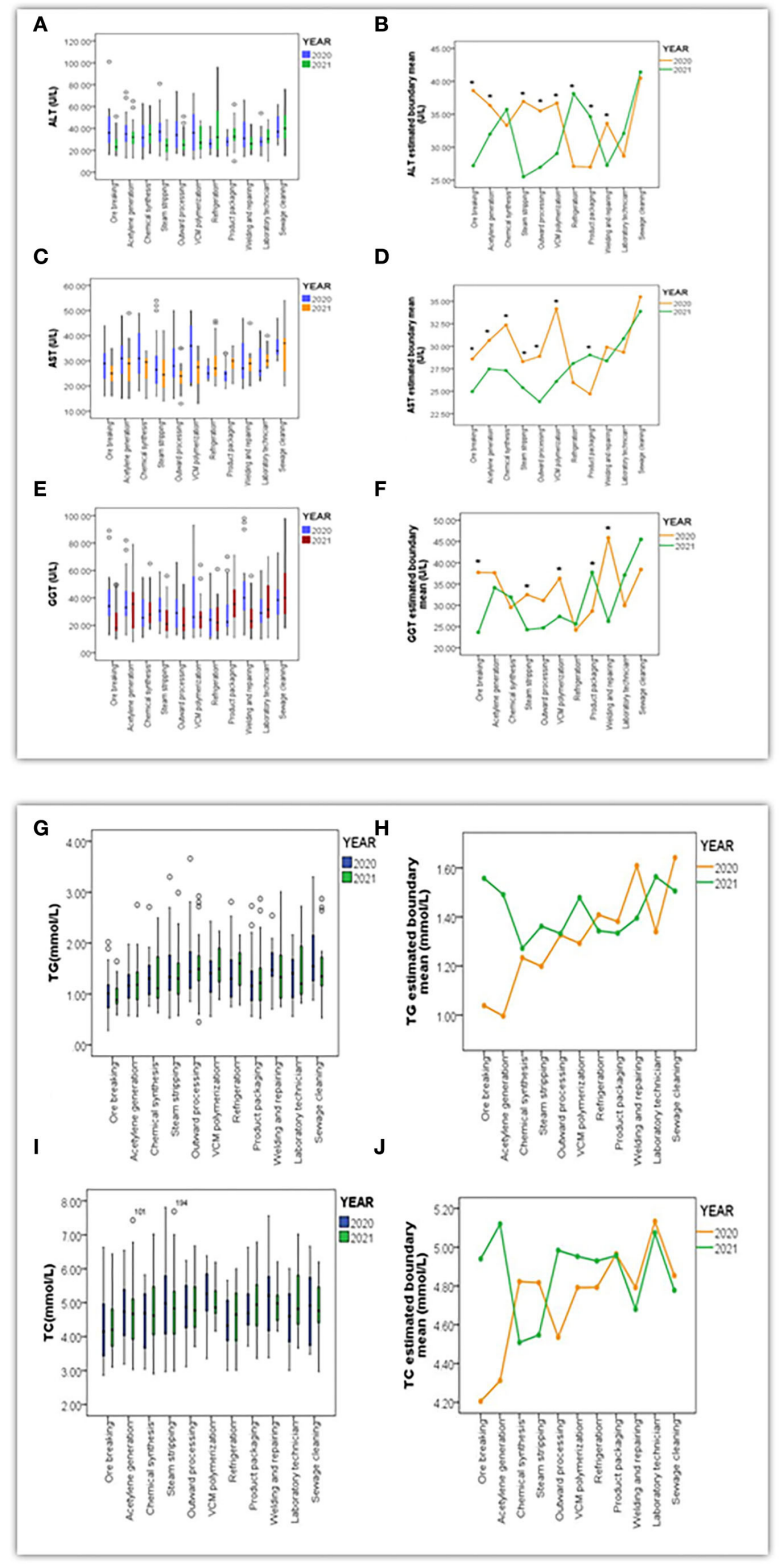
Box charts **(A,C,E,G,I)** presented the quantitative distribution extent of variables in ALT (U/L), AST (U/L), GGT (U/L), TG (mmol/L) and TC (mmol/L) throughout positions in Y between 2020 and 2021; Line Charts **(B,D,F,H,J)** indicated differentiation of estimated boundary mean for ALT, AST, GGT, TG, and TC under the bilateral interaction effect between year and position (*referred to *P* < 0.05 when differences of variables were significant).

**Figure 5:**
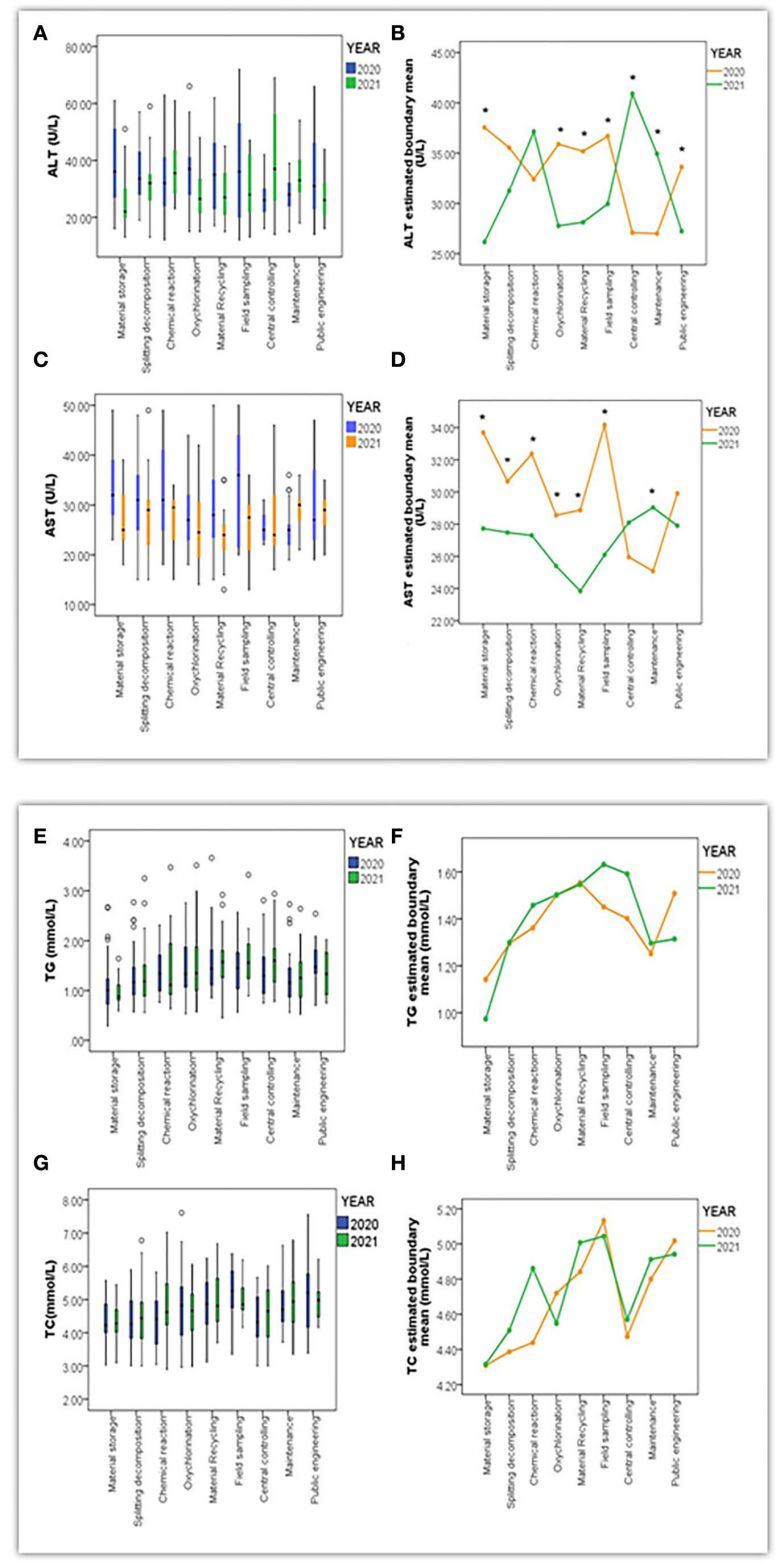
Box charts **(A,C,E,G)** presented the quantitative distribution extent of variables in ALT (U/L) and AST (U/L), TG (mmol/L), and TC (mmol/L) throughout positions in Z between 2020 and 2021. Line Chart **(B,D,F,H)** indicated differentiation of estimated boundary mean for ALT, AST, TG, and TC under a bilateral interaction effect between year and position (*referred to *P* < 0.05 when differences among variables were significant).

As the results above show, indicators such as ALT, AST, GGT, TG, and TC played pivotal roles in contributing to fatty liver and other hepatic symptoms. These differences still needed to be discovered in terms of what positions they could significantly affect. The box charts in Figures 4A,C,E,G,I, 5A,C,E,G illustrate the quantitative distribution differences of indicators toward positions in Y and Z between 2020 and 2021. In that regard, significant disparity toward ALT, AST, and GGT in Y involved ore breaking, steam stripping, VCM polymerization, outward processing, product packaging, welding, and repairing, while similar discrepancy toward ALT and AST in Z referred to material storage, chemical reactions, field sampling, oxychlorination, material recycling, and maintenance. Furthermore, by utilizing analysis of multivariate ANOVA analysis, we observed significant differences among variables such as ALT (*F* = 5.12, *P* < 0.001), AST (*F* = 3.31, *P* < 0.001), and GGT (*F* = 4.42, *P* < 0.001) in Y using a bilateral interaction for a year and position categories, but no such influence was found in TG (*F* = 0.68, *P* > 0.05) or TC (*F* = 0.80, *P* > 0.05). Specifically, the *LSD* test further indicated that positions of outward processing (*P* = 0.002, *P* = 0.026, *P* = 0.003), VCM polymerization (*P* = 0.011, *P* = 0.026, *P* = 0.020), steam stripping (*P* = 0.020, *P* = 0.010, *P* = 0.016), and product packaging (*P* = 0.027, *P* = 0.028, *P* = 0.011) demonstrated differences on ALT, AST, and GGT at the same time as compared to others, as shown in Figures 4B,D,F,H,J.

In contrast, this bilateral interaction in Z presented significant differences in ALT (*F* = 7.50, *P* < 0.001) and AST (*F* = 4.04, *P* < 0.001), and no such effect was found in TG (*F* = 0.64, *P* > 0.05) and TC (*F* = 0.58, *P* > 0.05). Positions of material storage (*P* = 0.004, *P* = 0.002), field sampling (*P* = 0.011, *P* = 0.004), oxychlorination (*P* = 0.003, *P* = 0.011), maintenance (*P* = 0.008, *P* = 0.033), and material recycling (*P* = 0.003, *P* = 0.009) were displayed simultaneous differences in ALT and AST as compared to other positions, as Figures 5B,D,F,H present.

Overall, it could be inferred that positions such as steam stripping, outward processing, VCM polymerization, and product packaging in Y had abnormal rate alterations in fatty liver and other hepatic symptoms that significantly differed in ALT, AST, and GGT simultaneously and that these positions also indicated a reduced risk assessment level alteration using the semi-quantitative comprehensive index model. By comparison, positions of material storage, oxychlorination, material recycling, and field sampling in Z significantly differed in ALT and AST with similar changes, as positions were not in line with risk assessment levels, as no significant changes were found before and after improvement. Besides, TG and TC were not critical variables to affect the fatty liver, especially when differences were statistically analyzed simultaneously under year and position classification.

## Discussion

Nowadays, techniques of acetylene hydrochlorination are still the predominant processes for VCM synthesis and PVC production, with the advantages of abundant resources, low investment, and high return. However, the replacement of ethylene oxychlorination has been overwhelming because of the disadvantages of high energy consumption and heavy contamination of the environment. The technique of ethylene oxychlorination still has a long way to go before it can substitute for the former one, given the requirement of high-tech equipment and enormous amounts of imported ethylene as the raw material (40). Under these circumstances, improvements in protective measures and technology upgrades are urgently required to facilitate development, innovation, environmental protection, and labor health. Thus, we were interested to discover whether improvement implementation could work effectively on positions' risk assessment levels and liver health status.

According to the studies at home or abroad, VCM and PVC dust were major hazards from others that imposed adverse effects on workers' health, and the correlation of eternal concentration with the related incidence of ASL and HCC has already been linked. For example, Pirastu, R (41) found that inhaled PVC dust (particularly with an aerodynamic diameter of <5 mm) may remain in the pulmonary interstitium for years and gradually release residual VCM, which may account for the neoplastic transformation of an epithelial cell. Facciolà et al. (42) discovered that some laboratory studies revealed the pathogenic role of PVC and revealed the link between exposure to PVC dust and both non-malignant and malignant lung disease. Despite the low reactivity, the number of surface area atoms per unit mass was high for PVC dust, greatly enhancing the surface area for chemical reactions with bodily fluids and tissue in direct contact, resulting in persistent inflammation that led to pulmonary fibrosis or even carcinogenesis. In an Italian cohort of 1,658 workers from a VCM/PVC plant in Porto Marghera (Venice, Veneto Region, Italy), Ugo Fedeli and Paolo Girardi found an increased risk of stomach cancer (SMR 1.53, CI 1.06–2.19) and a high rate of liver cancer (57 observed deaths; SMR 2.30, CI 1.78–2.99). Mortality from liver cancer was consistently increased through the follow-up: SMRs were 2.09 (1.33–3.27), 2.80 (1.79–4.39), and 2.15 (1.37–3.36) across subsequent calendar periods (1973–1999, 2000–2007, 2008–2017, with 19 observed liver cancer deaths in each period. Besides, out of 56 deaths from all causes observed among workers with cumulative exposure above 5,188 ppm-years, 12 (21%) were identified as primary liver cancer with clinical or histological confirmation, reporting six HCC and six ASL cases. The SMR for lung cancer was 1.73 (90% confidence interval 0.93–3.21) among “only baggers”; the ratio between the SMR for “only baggers” and that for “never baggers” was 2.31 (90% CI: 1.15–4.61) (43). In a re-analysis of mortality data from the same plant, with respect to the reference group (technicians and clerks), the lung cancer rate ratio was 3.13 (95% CI 0.96–10.28) in PVC baggers. In another case-control study nested in the same Porto Marghera cohort, 38 patients with a histological lung cancer diagnosis were compared with 224 controls without cancer. A logistic regression analysis showed an increase of 20% (odds ratio: 1.20; 95% CI: 1.07–1.35) in the risk of lung cancer for each additional year of work as a PVC packer, taking into account age and smoking. By excluding a potentially important source of bias, the adjustment for smoking strengthened the results of previous studies showing an increased risk for lung cancer among PVC baggers. Long-term exposure to high levels of PVC dust might cause pulmonary carcinogenesis through persistent alveolar inflammation, alveolar macrophage activation, and the release of growth factors (44).

Next, the demographic and occupational information in Y and Z indicated that the population distribution by gender between Y and Z was largely homogeneous, and VCM-exposed workers were under a heavy workload. Factors like long weekly working periods, frequent shift systems, sleep deprivation, or disorders might be potentially hazardous factors affecting physical health. The operation status of ventilation facilities had not reached its maximum, as temporary suspensions or fully broken systems were witnessed or described by workers on duty. It could be inferred that improving protective measures, especially the ventilation facilities, was necessary and urgent.

Afterward, improvements in protective measures in Y were implemented in 2021, mainly through the enhancement of ventilation and collection facilities as fresh air requirements and ventilation air changing rates at local plants was intensified so that ambient concentrations for VCM, PVC dust, and others were found to be declining, which could be a result of a stronger negative correlation between ventilation effects and ambient concentration in Y. On the contrary, the hazards of VCM, 1, 2DCE, and others in Z slightly declined due to improvements in sealing and airtight measures, but the magnitude of the correlation was not as significant as it was in Y. It could be inferred that improvements in protective measures in Y effectively limited hazard concentration levels, while that effect in Z was not prominent as natural ventilation was another main confounding factor that impacted air motion at workplaces.

However, it did not result in deleterious consequences from low-concentration VCM exposure, which could be ignored even if the ambient concentration was successfully reduced. More research found that low-concentration VCM exposure would induce health issues. For example, in a US cohort, based on 32 cases of HCC identified from death certificates, mortality rates did not increase, except for the highest quintile of cumulative exposure (≥2,271 ppm-years). However, after exposures were lagged by 30 years, HCC mortality significantly increased already in the 865–2,271 ppm-years class (45). In the European cohort of vinyl chloride workers, increased liver cancer risk (all types) with increasing exposure was confirmed in analyses restricted to subjects with cumulative exposure <1,500 ppm-years. In an Italian cohort, an approach based on non-parametric regression was adopted to model in continuous form the relationship between exposure and mortality, considering 31 confirmed HCC cases; HCC mortality rates were found to increase with cumulative VCM exposure already in the range below 2,000 ppm-years (43).

Moreover, for the sake of screening out typical positions that could encounter health risks under different techniques and discovering whether differences in risk levels before and after improvements in protective measures might exist, three semi-quantitative risk assessment models were applied. The results showed that the semi-quantitative comprehensive index model significantly differed in risk level alterations before and after improvements in Y (*Z* = 1.62, *P* = 0.011), and no such alterations were ever observed from models of ICMM and occupational hazards classification at workplaces. In that, assessment levels concerning ore breaking and acetylene generation declined to low risk in 2021 from medium risk in 2020, the risk of steam stripping, outward processing, VCM polymerization, welding, and repairing dropped to medium risk from high risk in 2020, and others like refrigeration (low), product packaging (low), and the laboratory technician (medium) remained unchanged before and after improvement, even if risk levels under exposure to Cl_2_, HCl, NaOH, and H_2_S all reduced to low risk in 2021 from medium ones in 2020. These may stem from models' advantages and limitations in terms of methodological principles. Concretely, the semi-quantitative comprehensive index model was originally converted from the risk assessment of Singapore model from OHRA and incorporated into the Chinese national guideline for occupational health risk assessment (GBZ 298-2017) with adjusted modifications. More than that, it developed its own comprehensive advantages by taking ambient concentration, protective measures, emergency rescue measures, and other semi-quantitative factors into account, which made risk levels more tightly bound with practical situations and more subjected to present alteration once hardware improvements or innovative changes were operated.

Moreover, the ICMM model was mainly evaluated through professional knowledge and working experience when determining hazard levels, leading to subjectivity and justification bias in the methodology (46). Thus, risks would usually be overestimated as long as workers were exposed to hazards that would cause severe harm under a longer working period. Conversely, the classification of occupational hazards (dust and chemical agents) at workplaces usually would be underestimated only if B assignments that referred to C_TWA_/OEL or C_M_/OEL were lower than 1 (B <1), then risk classifications turned out to be relatively harmless no matter what differences other weight factors could affect (47). Furthermore, their RR sequence among the three models was ordered from RR ICMM > RR semi-quantitative comprehensive index model > RR classification of occupational hazards at workplaces (dust and chemical agents) in China (*P* < 0.05). These results were supported by a similar study from Qiu liang Xu's (48) research, which stated that the EPA model achieved the highest RR [0.8 (0.2–1.0)], respectively, followed by the COSHH model [0.6 (0.6–1.0)], the Singaporean model [0.4 (0.2–0.8)], the Australian model [0.4 (0.2–0.6)]. The Romanian model [0.3 (0.3–0.4)] and the ICMM model [0.2 (0.2–0.8)] had the lowest RR. The order of RR among the six models was as follows: RR EPA > RR COSHH > RR Singaporean > RR Australian > RR Romanian > RR ICMM (*P* < 0.05), The Singaporean model was positively correlated with the other five models (*P* < 0.01), and their correlation coefficients were relatively greater than others, which could be attributed to its characteristics of compensating for shortcomings in quantitative and qualitative methods and giving relatively practical results by combining investigation data and standardized judgment. Above all, the semi-quantitative comprehensive index model was the most appropriate one among the two others for improvements in protective measures in a self-contrast pattern.

Subsequently, physical examination data between 2020 and 2021 were analyzed to discover whether differences among liver function indicators could be found. In fact, significant differences toward abnormal rates of fatty liver and other hepatic symptoms were only found in Y (*X*^2^ = 10.19, *P* < 0.001) between years, and no such effect was discovered among positions (*P* > 0.05), which indicated that there were changes in abnormal rates among positions before and after improvements on protective measures, but the low sample size for individual positions caused insignificance. Particularly, the majority of positions in Y and Z demonstrated a declining tendency on abnormal rates toward other hepatic symptoms from 2020 to 2021, in which the ones with relatively higher reduction rates involved in outward processing, product packaging, sewage cleaning, VCM polymerization, refrigeration, steam stripping in Y and material storage, chemical reactions, and splitting decomposition in Z, and no such apparent trend on the fatty liver was observed. It should be noted that the number of people in every position has mostly stayed the same from 2020 to 2021. It was unlikely to witness a significant alleviation of a series of organic liver lesions, such as hepatic cysts, intrahepatic calcification, and thickened intrahepatic echo, in a short interval phase of 1 year, unless there were new patients enrolled to substitute for the individuals with hepatic symptoms. They were further arranged for recuperation and position switching. In addition, to explore whether improvements in protective measures in Y and Z played a role in the reduction of abnormal rates of fatty liver and other hepatic symptoms, analyses of multiple linear regression and multivariate ANOVA were performed.

Results showed that in Y, the variable for males, ALT, AST, GGT, TG, and TC were factors contributing to fatty liver in Y in 2020. A similar effect was only seen in males, TG, and TC in 2021, and then variables of men, ALT, AST, and GGT were found to be essential to other hepatic symptoms, with only men left in effect in 2021. Meanwhile, in Z, the variables for men, TG and TC, which contained fatty liver, while males ALT and AST affected other hepatic symptoms in both 2020 and 2021. In addition, the male variable was the most significant factor among others to play a critical role in alterations toward liver ultrasound as demographic proportions in Y and Z were approximately five-fold and three-fold higher in men than in women. It was undeniable that the overwhelming proportion of males made a great contribution. TG and TC were critical variables to affect the fatty liver, but they were not when differences using a bilateral interaction between years and positions were analyzed. Combined with charts from Figures 3A,B, it is worth mentioning that positions such as steam stripping, outward processing, VCM polymerization, and product packaging in Y were ones with alterations toward abnormal rates in fatty liver and other hepatic symptoms that significantly differed in ALT, AST, and GGT at the same time, while material storage, oxychlorination, material recycling, and field sampling in Z were ones with similar changes that differed in ALT and AST simultaneously.

It could be inferred that, after improvements in protective measures (2021), variables such as ALT, AST, and GGT were no longer critical indicators to affect the fatty liver and other hepatic symptoms. When combined with the results of the on-site survey, it was possible to assume that the protective measures had improved in Y and Z and that those changes may have contributed to the positions' health improvement by practically reducing the disadvantages. However, no such alteration was seen from TG and TC to fatty liver, and it appeared that this improvement had no obvious influence on abnormal liver health in Z as indicators of TG, TC, ALT, and AST. For instance, ALT and AST normally exist within hepatocytes. They would be released into the bloodstream once impairment or cell death occurred. The ratio of AST/ALT was a common indicator to signify liver cell damage within normal ranges. The extent of damage could be judged to be mild when the ratio was lower than 1. It might uncover a much more serious level involved in severe hepatitis, cirrhosis, and even HCC when the ratio exceeds 1. It also would be helpful to diagnose alcoholic liver disease, especially when the ratio was extremely higher than 2. Serum transaminases might be partially related to nonalcoholic fatty liver disease (NAFLD), which may eventually progress to liver fibrosis, cirrhosis, and cancer (49). For instance, Lang et al.'s (50) studies also found that joint action between VCM and HFD significantly enhanced liver disease and further resulted in some inflammatory foci and alterations of ALT and AST in circular blood, which were sufficient to exacerbate experimental NAFLD, as VCM did cause the liver to be more susceptible to damage from a secondary insult by decreasing mitochondrial function. Notably, serum transaminases of those with non-alcoholic steatohepatitis (TASH) were not altered with respect to healthy chemical workers. The consequences of current high VCM exposures may not always be reversible after exposure has been withdrawn and may further evolve into progressive liver injury and fibrosis (51, 52). The study conducted on clinical data and biological specimens from Louisville, Kentucky, demonstrated the prevalence of TASH, a liver pathology, in highly exposed VCM plant workers because of its noncancerous pathophysiology. TASH is a progressive form of nonalcoholic fatty liver disease (NAFLD). NAFLD is a spectrum of liver disorders ranging from lipid accumulation (steatosis) and hepatic inflammation (steatohepatitis) to the presence of fibrosis and cirrhosis (53, 54).

In this regard, it could be inferred that alterations toward fatty liver and other hepatic symptoms among positions before and after improvements in protective measures could be partially caused by corresponding changes of ALT, AST, and GGT in typical positions in Y and Z, but their evidence for specific significance to fatty liver and other hepatic symptoms and relationship with improvements in protective measures in Y and Z needs further exploration.

## Limitation

Several limitations prevented us from conducting systematic research on the relationship between improvements in protective measures and the health problems caused by VCM and other hazards. (1) This research could not connect health indicators with position classification for failure on collection of position's classification from the physical examination process for consecutive years, the available data are not enough to verify the relationship between improvement on protective measures and liver health status. (2) In addition to the ventilation effect at indoor plants, we missed the effect of the natural ventilation requirement on ambient concentration alterations as numerous devices or facilities were placed outdoors; (3) General maintenance for sealing and airtight devices, valves, pipes, or sampling facilities were found to be improved in 2021, which radically inhibited evaporation and effusion of organic solvents or industrial dust, but we failed to verify their enhancement through the collection of quantitative data; (4) The very important catalyst HgCl_2_ failed to be brought into detection as it existed in a solid pattern during the production process, and mercury-containing wastewater was not our primary purpose.

## Conclusion

This study selected two factories with different techniques for VCM and PVC synthesis to evaluate the effect of improving protective measures in 2020 on alterations of health risk levels and liver function indicators. Severe conclusions could be drawn from the following: (1) Improvements in protective measures in Y and Z contributed to the reduction of ambient concentration at workplaces through the promotion of local ventilation effects and sealing airtight measures; (2) the semi-quantitative comprehensive index model is appropriate for evaluating risk level alterations before and after improvements on measures in a self-contrast pattern; and (3) alterations toward fatty liver and other hepatic symptoms among positions before and after improvements in protective measures could be partially caused by corresponding changes in ALT, AST, and GGT.

## Data availability statement

The original contributions presented in the study are included in the article/supplementary material, further inquiries can be directed to the corresponding author.

## Ethics statement

The studies involving human participants were reviewed and approved by Medical Ethical Review Committee of National Institute for Occupational Health and Poison Control, Chinese Center for Disease Control and Prevention. The patients/participants provided their written informed consent to participate in this study. Written informed consent was obtained from the individual(s) for the publication of any potentially identifiable images or data included in this article.

## Author contributions

Writing for original draft preparation: YD. Conception or design of the work: YD, XingW, WH, and MY. On-site survey or field investigation work: YD, XingW, WH, HB, XinW, NK, FH, and SZ. Questionnaire, data acquisition, analysis, or interpretation of data for the work: YD, HB, XinW, NK, FH, and SZ. Revising critically for important intellectual content: MY. All authors have read and agreed to the published version of the manuscript.

## Funding

This work was funded by the Occupational Population Survey in Key Industries, National Institute for Occupational Health and Poison Control, Chinese Center for Disease Control and Prevention (Grant No. 131031109000210004).

## Conflict of interest

The authors declare that the research was conducted in the absence of any commercial or financial relationships that could be construed as a potential conflict of interest.

## Publisher's note

All claims expressed in this article are solely those of the authors and do not necessarily represent those of their affiliated organizations, or those of the publisher, the editors and the reviewers. Any product that may be evaluated in this article, or claim that may be made by its manufacturer, is not guaranteed or endorsed by the publisher.
